# Genome-wide prediction and prioritization of human aging genes by data fusion: a machine learning approach

**DOI:** 10.1186/s12864-019-6140-0

**Published:** 2019-11-09

**Authors:** Masoud Arabfard, Mina Ohadi, Vahid Rezaei Tabar, Ahmad Delbari, Kaveh Kavousi

**Affiliations:** 1 Department of Bioinformatics, Kish International Campus University of Tehran, Kish, Iran; 2 Laboratory of Complex Biological Systems and Bioinformatics (CBB), Department of Bioinformatics, Institute of Biochemistry and Biophysics (IBB), University of Tehran, Tehran, Iran; 3 Iranian Research Center on Aging, University of Social Welfare and Rehabilitation Sciences, Tehran, Iran; 4 Department of Statistics, Faculty of Mathematical Sciences and Computer, Allameh Tabataba’i University, Tehran, Iran

**Keywords:** Genome-wide, Prioritization, Human aging genes, Positive unlabeled learning, Machine learning

## Abstract

**Background:**

Machine learning can effectively nominate novel genes for various research purposes in the laboratory. On a genome-wide scale, we implemented multiple databases and algorithms to *p*redict and *p*rioritize the *h*uman *a*ging *ge*nes (PPHAGE).

**Results:**

We fused data from 11 databases, and used Naïve Bayes classifier and positive unlabeled learning (PUL) methods, NB, Spy, and Rocchio-SVM, to rank human genes in respect with their implication in aging. The PUL methods enabled us to identify a list of negative (non-aging) genes to use alongside the seed (known age-related) genes in the ranking process. Comparison of the PUL algorithms revealed that none of the methods for identifying a negative sample were advantageous over other methods, and their simultaneous use in a form of fusion was critical for obtaining optimal results (PPHAGE is publicly available at https://cbb.ut.ac.ir/pphage).

**Conclusion:**

We predict and prioritize over 3,000 candidate age-related genes in human, based on significant ranking scores. The identified candidate genes are associated with pathways, ontologies, and diseases that are linked to aging, such as cancer and diabetes**.** Our data offer a platform for future experimental research on the genetic and biological aspects of aging. Additionally, we demonstrate that fusion of PUL methods and data sources can be successfully used for aging and disease candidate gene prioritization.

## Background

Prior understanding of the genetic basis of a disease is a crucial step for the better diagnosis and treatment of the disease [1]. Machine learning methods help specialists and biologists the use of functional or inherent properties of genes in the selection of candidate genes [2]. Perhaps the question that is posed to researchers is why all research is aimed at identifying pathogenic rather than non-pathogenic genes. The answer may lie in the fact that genes introduced as non-pathogens may be documented as disease genes later on.

Biologists apply computation, mathematics methods, and algorithms to develop machine learning methods of identifying novel candidate disease genes [3]. Based on the principle of “guilt by association”, similar or identical diseases share genes that are very similar in function or intrinsic properties, or have direct physical protein-protein interactions [4]. Most methods of predicting candidate genes employ various biological data, such as protein sequence, functional annotation, gene expression, protein-protein interaction networks, regulatory data and even orthogonal and conservation data, to identify similarities with respect to the principle of association based on similarity [5]. These methods are categorized as unsupervised, supervised, and semi-supervised [6]. Unsupervised methods cluster the genes based on their proximity and similarity to the known disease genes, and rank them by various methods. Supervised methods create a boundary between disease genes and non-disease genes, and utilize this boundary to select candidate genes. Several studies have been performed to address different aspects of the methodology and have expanded the use of various methods and tools [3, 7–12].

The tools that are available for candidate gene prioritization can be classified with respect to efficiency, computational algorithms, data sources, and availability [13–15]. Available prioritization tools can be categorized into specific and general tools [16]. Specific tools are used to prioritize candidate genes associated with a specific disease. In these methods, information related to a specific tissue involved in the disease or other information related to the disease is employed. General tools can be applied for most diseases, and various data sources are often used in these tools. Gene prioritization tools can be divided into two types of single-species and multi-species. Single-species tools are only usable for a specific species, such as human or mouse. Multi-species tools have the ability to prioritize candidate genes in several different species. For example, the ENDEAVOR software can prioritize the candidate genes in six different species [17]. With respect to computational algorithms, candidate prioritization tools are primarily divided into two groups of complex network-based methods and similarity-based methods [5]. The inevitable completeness and existence of errors in biological data sources necessitate fusion of multiple data sources [18]. Most gene targeting methods, therefore, use multiple data sources to improve performance.

The purpose of this study was to design a machine to identify and prioritize novel candidate aging genes in human. We examined the existing methods of identifying human non-aging (negative) genes in the machine learning techniques, and then made a binary classifier for predicting novel candidate genes, based on the positively and negatively learned genes. Gene ranking was based on the principle of the similarity among positive genes through “guilt by association”. Thus, across the unlabeled genes, genes that were less similar in respect with the known genes were employed as negative sample.

## Results

The three positive unlabeled learning (PUL) algorithms, Naïve Bayes (NB), Spy, and Rocchio-SVM, were used to evaluate the underlying data, and to compare them to the eight datasets introduced with respect to performance. All samples of a class with a higher frequency were unlabeled. We applied the algorithm to predict the labels. These methods utilize a two-step strategy and are intended to extract a reliable negative sample from the main data (Table 1).

**Table 1:** Datasets used to evaluate reliable negative sample extraction algorithms

Number of instances	Number of attributes	Data set names
756	754	Parkinson’s Disease Classification Data Set [19]
345	7	Liver Disorders Data Set [20]
1024	10	Cloud Data Set [21]
351	34	Ionosphere Data Set [22]
19,020	11	MAGIC Gamma Telescope Data Set [23]
961	6	Mammographic Mass Data Set [24]
569	32	Breast Cancer Wisconsin (Diagnostic) Data Set [25]
208	60	Connectionist Bench (Sonar, Mines vs. Rocks) Data Set [26]

We also randomly selected 70% of the positive samples as the training set, and the remainder as the test set. To determine the classifier, positive and negative samples were equally selected to ensure that the classifier did not have any bias at the training step. Therefore, we compared the three algorithms with eight data sources extracted from the UCI database (Additional file 1).

Comparison of the parameters of the three algorithms for all data sets revealed similar results in F_measure. For example, in data set 1, the precision of the Roc-SVM method, (approximately 2–3%,) was better than those of the other two methods. However, the recall of the NB method (approximately 4–6%,) was better than those of the other two methods, and Roc-SVM method had a lower false positive rate than that of the other two methods (Table 2). In addition, comparison between the parameters of the three algorithms for data set 2, revealed that the precision of the NB method was better than that of the other two methods, the recall SPY method was 5% better than that of the other two methods, and the NB method had a lower false positive rate than that of the other two methods. Therefore, none of the methods had an absolute superiority. Since the results were very similar, the output of the three methods was combined.

**Table 2:** Performance evaluation of the reliable negative sample extraction algorithms

Data set	Algorithm	FPR%	FNR%	Precision %	Recall %	F_measure %
Parkinson’s Disease	NB	37.25	4.57	95.43	89.78	92.52
	SPY	8.70	16.11	97.42	83.89	90.15
	Roc-SVM	6.52	15.00	98.08	85.00	91.07
Liver Disorders	NB	17.65	5.71	73.33	94.29	82.50
	SPY	36.14	0	40.00	100	57.14
	Roc-SVM	31.33	5.00	42.22	95.00	58.46
Cloud	NB	18.88	7.93	84.83	92.07	88.30
	SPY	9.52	14.92	92.77	85.08	88.76
	Roc-SVM	6.32	16.51	96.72	83.49	89.62
Ionosphere	NB	47.62	8.33	88.51	91.67	90.06
	SPY	26.32	6.98	94.12	93.02	93.57
	Roc-SVM	33.33	8.89	94.25	91.11	92.66
MAGIC Gamma Telescope	NB	10.49	44.44	68.18	55.56	61.22
	SPY	17.88	36.22	53.88	63.78	58.42
	Roc-SVM	6.68	47.18	77.65	52.82	62.87
Mammographic Mass	NB	7.25	33.72	85.07	66.28	74.51
	SPY	11.96	10.00	62.07	90.00	73.47
	Roc-SVM	1.95	28.57	94.34	71.43	81.30
Breast Cancer Wisconsin	NB	13.85	12.26	91.18	87.74	89.42
	SPY	9.09	10.48	94.00	89.52	91.71
	Roc-SVM	22.50	22.14	91.89	77.86	84.30
Connectionist Bench (Sonar, Mines vs. Rocks)	NB	13.85	12.26	91.18	87.74	89.42
	SPY	16.67	7.69	80.00	92.31	85.71
	Roc-SVM	22.50	22.14	91.89	77.86	84.30

**Fig. 1:**
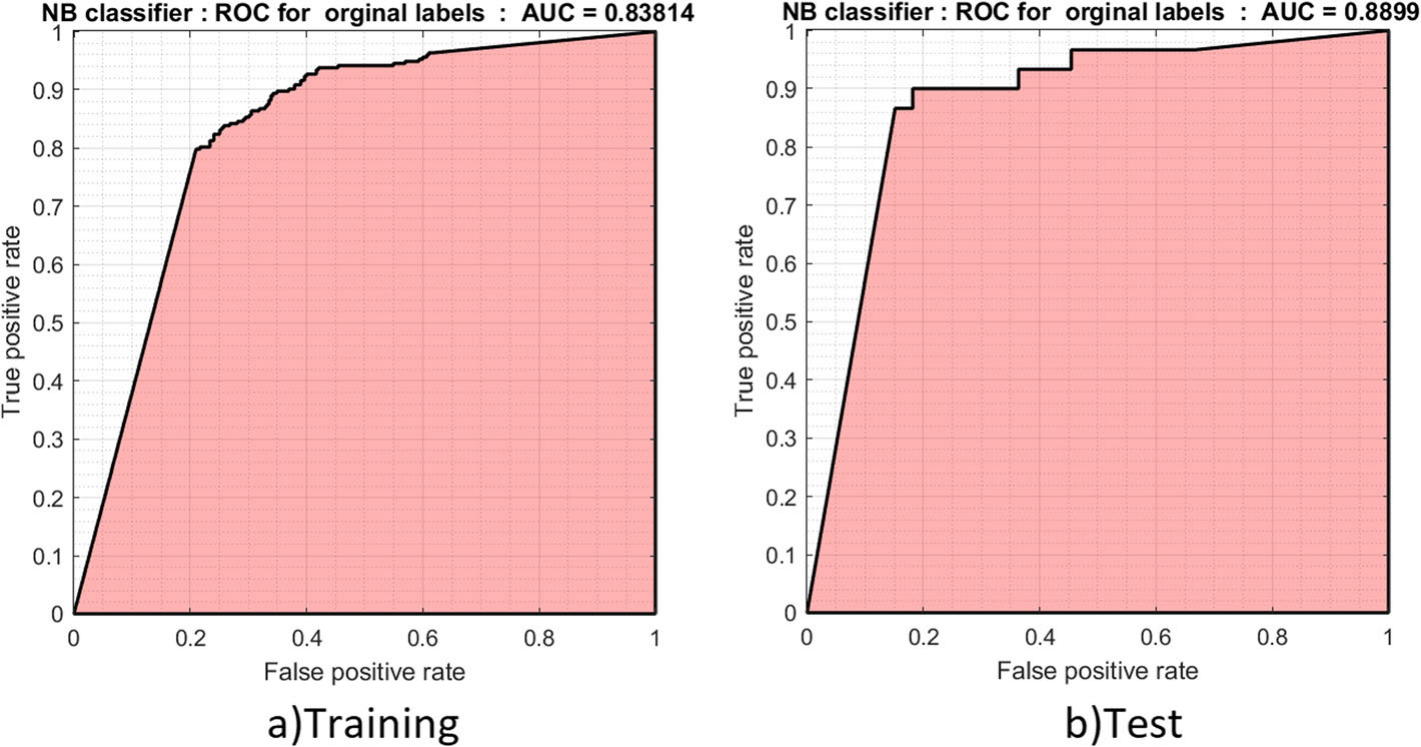
ROC curves. ROC was performed to evaluate the performance of the Naïve Bayes model at the training and test steps, which resulted in similar values for both curves

The three PUL algorithms were applied to extract reliable negative samples and to compare them with respect to performance. In this algorithm, only 303 positive samples were given as input, which enabled extraction of reliable negative samples from the remaining data. Subsequently, from the positive and negative data, a new classifier was trained to identify novel candidate genes to be utilized for prioritization and ranking. A total of 328 negative genes were extracted from each positive and negative gene, with a threshold of 11 replicates per negative gene (Additional file 2), and the Naïve Bayes binary classifiers were trained in a 10-fold cross-validation (Table 3). Additional file 2 contains results for all thresholds. The ROC chart for training and test data is shown in Fig. 1.

**Table 3:** Model performance evaluation by Naïve Bayes on the aging data

	Precision %	Recall %	F measure %	Accuracy %	AUC %
Train	80.78	76.95	78.81	78.52	83.81
Test	87.09	81.82	84.37	84.13	88.99

We trained multiple binary classifiers using all features in the positive genes and reliable negative data to compare the NB classifier to other classifiers. We investigated the performance of binary SVM [27], NB, and libD3C [28] classifiers in the dataset with 10-Fold cross validation, using Weka [29]. All classifiers had similar performance in the main data set (Table 4).

**Table 4:** Performance evaluation comparison by multiple binary classifier in the aging data

	TP rate %	FP rate%	Precision %	Recall %	F measure %	AUC %
SVM	80	21.1	82	80	79.6	79.5
libD3C	85.1	15.3	85.3	85.1	85	91.9
NB	81.1	19.7	82.4	81.1	80.9	86

A major challenge in classification is to reduce the dimensionality of the feature space. Some methods, such as PCA, are linear combinations of the original features. In this research, we investigated the PCA method in the final model, which eliminated some of the original input features and retained a minimum subset of features that yielded the best classification performance. In addition, the feature selection technique was used to select the best subset of features that were satisfying to the model in respect with the subset of the main features. A fixed number of top ranked features were selected to design a classifier. A suitable technique for feature selection is minimal-redundancy-maximal-relevance (mRMR) [30]. We also used mRMR for feature selection in the main data, and then compared multiple binary classifiers in the positive and reliable negative genes. We investigated the top 500 ranked features that were extracted from the mRMR tool to compare the classifiers. All of the selected classifiers yielded acceptable results (Table 5).

**Table 5:** Performance evaluation comparison by multiple binary classifier in the aging data after feature selection

	TP rate %	FP rate%	Precision %	Recall %	F measure %	AUC %
SVM	83.5	17.1	84.2	83.5	83.4	83.2
libD3C	84.6	15.7	84.8	84.6	84.6	92.3
NB	81.9	18.5	82.1	81.9	81.9	86.8

Model accuracy assurance is very difficult when the model applied to a separate test suite includes positive and unlabeled samples. This challenge is critical in instances which lack negative sample. Thus, we compared the evaluation metric with the data. We generated data for all 10 models in the training section to predict the residual genes, and extracted the genes that were identified by the 10 models as positive genes, yielding a total of 3531 final candidate genes.

To compare the output of the method with the known tools for prioritizing the genes, the output of the model was compared with two softwares, Endeavor [17] and ToppGene [31], in the seed genes.

(the list of seed genes in the form of K-Fold with K = 3 was utilized for the mentioned tools). Two metrics for comparing the tools with the proposed model were considered. The first metric calculated the average ranking for the seed genes, and the second metric determined the number of seed genes on the lists as 10, 50, 100, 500, and 1000.

A tool that had more seed genes at the top of the list and a lower average rating compared with the remaining tools, received a higher ranking. Table 6 shows the output of the tools and the PPHAGE method for determining the number of test genes on the known lists. Table 7 shows the output of tools and the PPHAGE method for the average rank score on different lists.

**Table 6:** Number of detected seed genes in comparison to the output of tools

Tools	Rank	Fold1	Fold2	Fold3
Endeavour	< 10	1	0	1
	< 50	2	0	2
	< 100	4	1	2
	< 500	11	12	17
	< 1000	24	25	25
ToppGene	< 10	2	0	1
	< 50	11	0	2
	< 100	16	1	2
	< 500	44	12	17
	< 1000	62	25	25
PPHAGE	< 10	2	2	0
	< 50	7	4	5
	< 100	12	12	9
	< 500	50	35	38
	< 1000	66	61	67

**Table 7:** Average rank of the seed genes in comparison to the output of tools

	Fold1	Fold2	Fold3
Endeavour	1851	1918	1877
ToppGene	926	849	1024
PPHAGE	833	919	930

The top 25 genes that received the highest weight among all candidate aging genes (Table 8), were validated in a number of instances, based on experimental evidence, age-related diseases, and genome-wide association studies (GWAS). A list of all candidate positive aging genes is provided in Additional file 3.

**Table 8:** The top 25 human candidate aging genes

Rank	Gene symbol	Relevance	Reference	Database reference
1	*NAP1L4*	Nucleosome Assembly	[32, 33]	
2	*CCNI* *(CYC1)*	Parkinson Disease	[34]	BEFREE
3	*RPL3*	Ribosomal Protein	[35]	
4	*FZD5*	Alzheimer’s Disease	[36]	BEFREE
5	*BRD2*	Diabetes Mellitus, Non-Insulin-Dependent Osteoporosis, Postmenopausal Colorectal Cancer	[37–40]	BEFREE
6	*ATP8A2*	ATPase Phospholipid Transporting	[41]	
7	*SRSF11*	Serine And Arginine Rich Splicing Factor	[42]	
8	*BBIP1*			
9	*IL10*	Cardiovascular Diseases Diabetes Mellitus, Non-Insulin-Dependent Colorectal Cancer Atherosclerosis Parkinson Disease Alzheimer’s Disease Arthritis Heart failure	[43, 44] [45–47] [48, 49] [50, 51] [52–54] [55–57] [58–60] [61–63]	CTD_human RGD LHGDN BEFREE HPO
10	*FYCO1*	Cataract, autosomal recessive congenital 2 Cataract	[64, 65]	UNIPROT GENOMICS_ENGLAND HPO CTD_human
11	*PSMB2*			
12	*NSF*	Parkinson Disease	[66–70]	GWASDB GWASCAT BEFREE
13	*OAZ1*			
14	*ZFP36L1*			
15	*PCLO*	Diabetes Mellitus, Non-Insulin-Dependent	[71]	BEFREE
16	*GAB2*	Alzheimer’s Disease Colorectal Cancer Osteopetrosis	[72–75] [76, 77] [78]	BEFREE GWASDB GWASCAT
17	*QKI*	Coronary heart disease Colorectal Cancer	[79]	BEFREE UNIPROT
18	*ZNF638*			
19	*RGS3*			
20	*XPO6*			
21	*ATP8B1*	Colorectal Cancer	[80]	BEFREE
22	*ITM2C*			
23	*RBFOX1*	Heart failure Colorectal Cancer	[81] [82]	BEFREE
24	*DLC1*	Colorectal Cancer Hereditary Diffuse Gastric Cancer Coronary heart disease Increased gastric cancer	[83] [84] [85]	BEFREE CTD_human HPO
25	*MVK*	Arthritis Cataract		HPO HPO

## Discussion

On a genome-wide scale, we used three PUL methods to create a method for the isolation of human aging genes from other genes. The combined use of several methods as a fusion of their output was advantageous over using one single method.

Following are examples of the identified genes and experimental or GWAS link between these genes and aging. On the list of the 25 top genes, *NAP1L4* encodes a member of the nucleosome assembly protein (NAP) family, which interacts with both core and linker histones, and shuttles between the cytoplasm and nucleus, suggesting a role as histone chaperone. Histone protein levels decline during aging, and dramatically affect chromatin structure. Remarkably, the lifespan can be extended by manipulations that reverse the age-dependent changes to chromatin structure, indicating the pivotal role of chromatin structure in aging [32]. In another example, gene expression of *NAP1L4* increases with age in the skin tissue [33]. Findings of GWAS link a number of the identified genes to age-related disorders, such as *GAB2* and late onset Alzheimer’s disease [86], and *QKI* and coronary heart disease/myocardial infarction [79]. Interestingly, GWAS reports also link *QKI* to successful aging [87].

*RPL3* encodes a ribosomal protein that is a component of the 60S subunit. The encoded protein belongs to the L3P family of ribosomal proteins, and is increased in gene expression during aging of skeletal muscle [88]. In another example, *FZD5* is involved in prostate cancer, which is the most common malignancy in older men. *ATP8A2* is another gene subject to deterioration and loss of function over time. *RYR2* (Additional file 3) encodes a ryanodine receptor found in cardiac muscle sarcoplasmic reticulum. Mutations in this gene are associated with stress-induced polymorphic ventricular tachycardia and arrhythmogenic right ventricular dysplasia and methylation analysis of CpG sites in DNA from blood cells showed a positive correlation between *RYR2* and age [89]. In additional examples, differential expression with age was identified in *BCAS3*, *TUFM* and *DST* in the skin [33]. Gene expression revealed a significant increase in the expression of hippocampal *TLR3* from elderly (aged 69–99 years old) compared to cells from younger individuals (aged 20–52 years old) [90]. Similarly, differential expression with age was identified in *RORA* in the adipose tissue [33].

In order to investigate the implication of the identified candidate genes in aging, we conducted a comprehensive analysis of 330 human pathways in the KEGG. Each of the pathways was examined in the seed and candidate genes, and direct association was detected in a number of instances. For example *IL10* activates *STAT3* in the FOXO signaling pathway. In another example, *GAB2* has a regulatory role for *PLCG2* in the osteoclast differentiation pathway, as well as an activating role in the chronic myeloid leukemia pathway. Likewise, *FOS* is an expression target for *IL10* in the T cell receptor signaling pathway.

Enrichment analysis was performed using the Enrichr tool, based on the candidate genes and the negative genes [91] to examine whether the candidate and negative genes were correctly selected in respect with aging. The analysis of candidate genes was performed on 3531 genes from the rest of the test genes (i.e. excluding the positive seed and reliable negative genes). Most diseases that were associated with the candidate genes were diseases that occur with aging (e.g. colorectal cancer and diabetes) (Table 9).

**Table 9:** Indicative diseases associated with the candidate aging genes

Index	Name	*P*-value	Adjusted *p*-value	Z-score	Combined score
1	Colorectal cancer	1.43e-08	0.000001256	−1.94	35.07
2	Leukemia	6.71e-07	0.00002953	−1.64	23.32
3	Breast_cancer	0.000009246	0.0002357	−1.45	16.76
4	Diabetes	0.00002362	0.0002986	−0.92	9.85
5	Anemia	0.00002185	0.0002986	−0.9	9.68
6	Cardiomyopathy	0.00002757	0.0002986	− 0.59	6.23

**Fig. 2:**
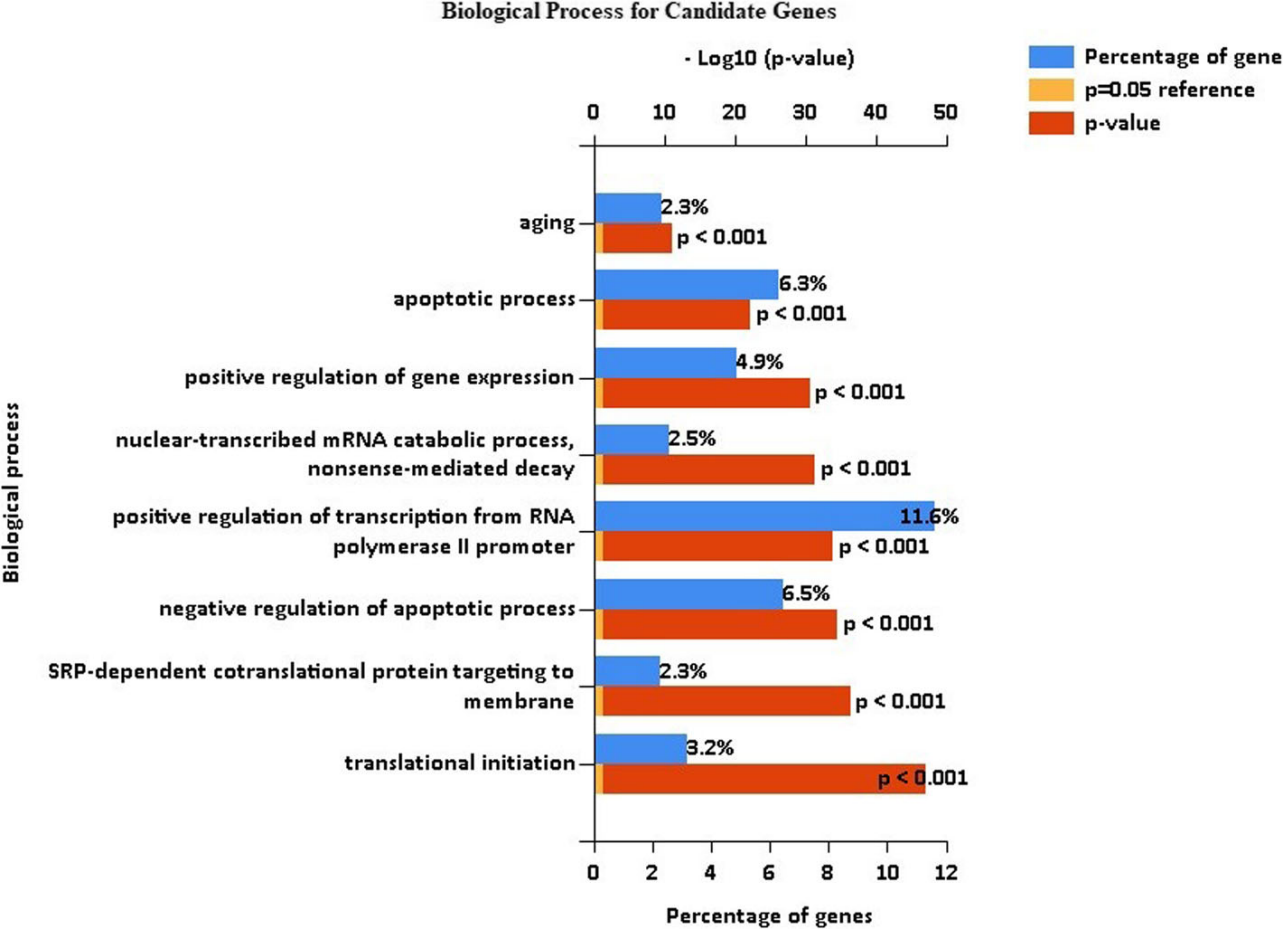
Significant biological processes associated with the candidate aging genes

Ontology analysis of the candidate genes was performed by FUNRICH [92] (Fig. 2), which revealed enrichment for the aging process and apoptosis. A list of all biological processes associated with the candidate aging gene is provided in Additional file 4.

In the analysis of the enriched biological pathways, using Enrichr (Table 10), cancer pathways had the highest score. Interestingly, viral pathways (e.g. EBV and HSV) were enriched in the positive aging genes compartment, which is in line with the previously reported immunosenescence and activation of such viruses as a result of aging [93] .A list of all biological pathways of the candidate genes extracted by FUNRICH is provided in Additional file 5.

**Table 10:** Indicative biological pathways associated with the candidate aging genes

Index	Name	*P*-value	Adjusted *p*-value	Z-score	Combined score
1	Pathways in cancer_Homo sapiens_hsa05200	4.07e-41	1.19e-38	−2.11	196.21
2	Proteoglycans in cancer_Homo sapiens_hsa05205	1.91e-31	2.78e-29	−1.99	140.58
3	Epstein-Barr virus infection_Homo sapiens_hsa05169	3.24e-30	3.15e-28	−1.9	128.92
4	Endocytosis_Homo sapiens_hsa04144	1.19e-28	8.70e-27	−1.89	121.38
5	Regulation of actin cytoskeleton_Homo sapiens_hsa04810	4.30e-26	2.51e-24	−1.82	106.42
6	HTLV-I infection_Homo sapiens_hsa05166	1.01e-25	4.21e-24	−1.79	103.2
7	Protein processing in endoplasmic reticulum_Homo sapiens_hsa04141	7.55e-26	3.68e-24	−1.69	98.04
8	Herpes simplex infection_Homo sapiens_hsa05168	1.24e-25	4.54e-24	−1.61	92.36
9	PI3K-Akt signaling pathway_Homo sapiens_hsa04151	1.79e-22	4.96e-21	−1.83	91.82
10	Focal adhesion_Homo sapiens_hsa04510	1.12e-22	3.63e-21	−1.72	86.98

**Fig. 3:**
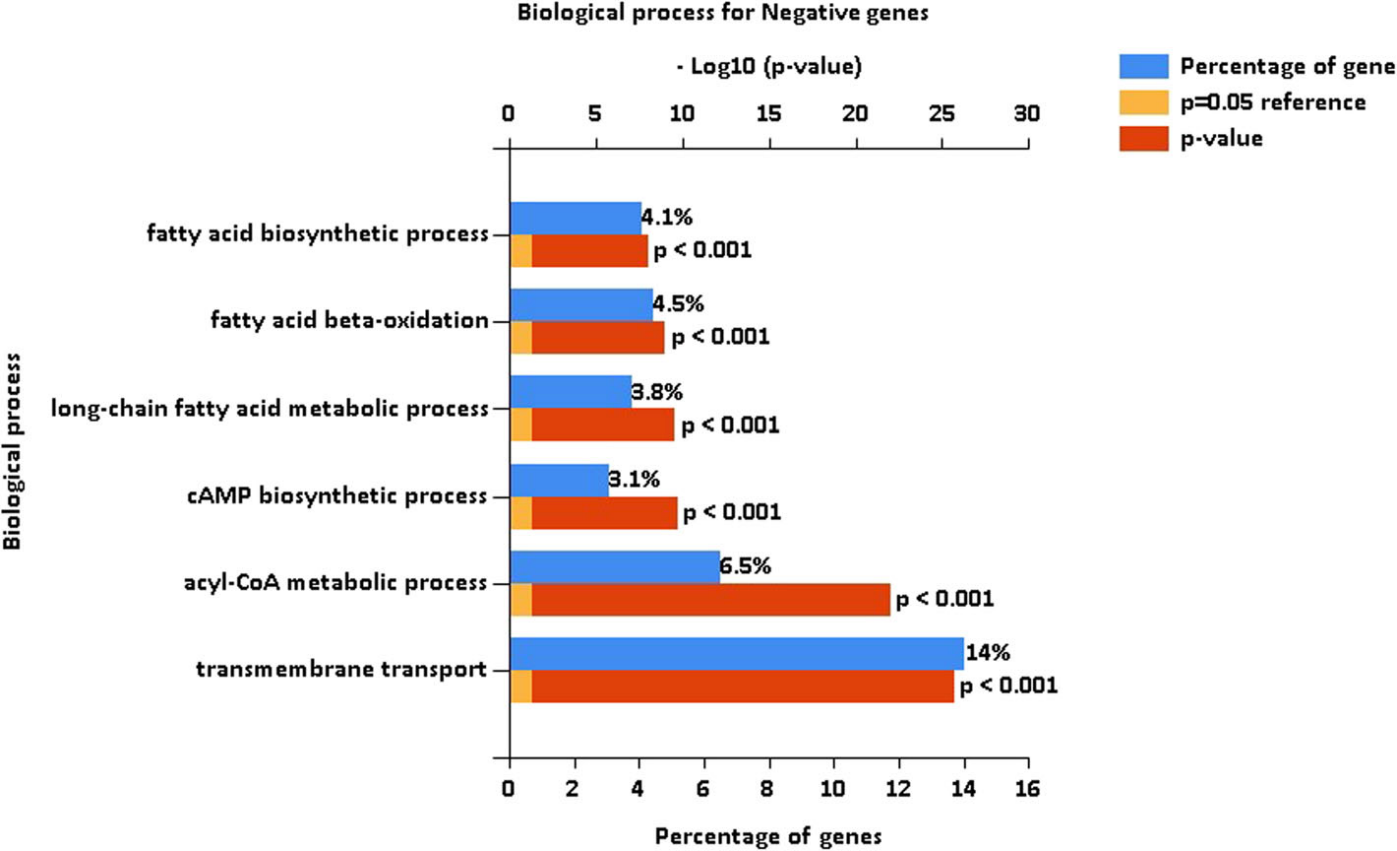
Significant biological processes associated with the reliable negative genes

No specific age-related diseases were detected for the identified negative genes (Table 11), which supports the validity of the model training used. Ontology analysis of the reliable negative genes (Fig. 3), which was also performed by FUNRICH, revealed that most of the extracted processes had a general role in all cells and could not be related to specific aging processes. Analyzing the biologic pathways in the negative genes indicated pathways that were predominantly unrelated to the aging processes.

**Table 11:** Indicative diseases associated with the reliable negative genes

Index	Name	*P*-value	Adjusted *p*-value	Z-score	Combined score
1	Cardiomyopathy,_dilated	0.01658	0.2321	−1.69	6.93
2	Cardiomyopathy	0.03134	0.2416	−1.61	5.57
3	Zellweger_syndrome	0.01588	0.2321	−1.06	4.41
4	Dystonia	0.03451	0.2416	−0.37	1.25

Based on the principle that similar disease genes are likely to have similar characteristics, some machine learning methods have been employed to predict new disease genes from known disease genes. Previous approaches developed a binary classification model that used known disease genes as a positive training set and unknown genes as a negative training set. However, the negative sets were often noisy because unknown genes could include healthy genes and positive collections. Therefore, the results presented by these methods may not be reliable. Using computational machine learning methods and similarity metrics, we identified reliable negative samples, and then tested the samples using a two-class classifier to identify novel positive aging genes in human.

## Conclusion

We implemented 11 databases and several machine learning methods to rank the entire human genes, and predicted and prioritized over 3,000 novel candidate age-related genes based on significant ranking scores. These genes were supported by biological, ontology, and disease enrichment analyses. Future experimental research is warranted to verify the significance of the identified genes in human aging.

## Methods

### Algorithms

**Fig. 4:**
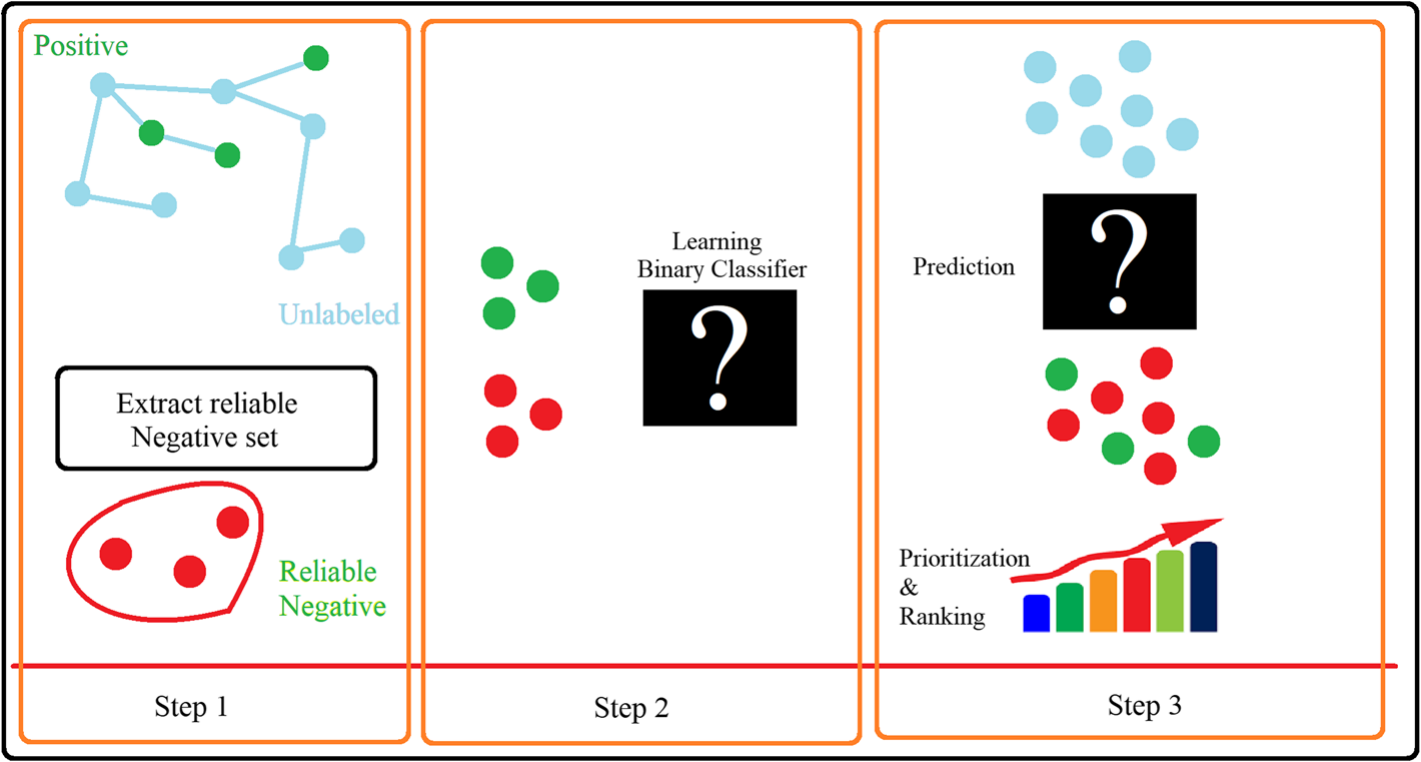
The overall learning scheme based on positive and unlabeled samples, and extraction of reliable negative samples (step 1), construction of the binary Classifier (step 2), and prediction and prioritization of candidate genes (step 3)

A classification method that is referred to as PUL is a similarity-based algorithm, in which reliable negative samples are extracted from unlabeled data. In addition, a binary classifier can be designed and used to identify the candidate genes (Fig. 4). Likewise, some methods identify reliable negative samples from unlabeled data, which are divided into three general categories: The first category has a two-stage strategy that runs a supervised algorithm on the data, by selecting reliable negative samples from within unlabeled instances [94]. The second category estimates the probability of positive samples by weighting positive and unlabeled data. The third category considers unlabeled data as negative samples with noise.

In this paper, a two-stage strategy was used to find a reliable negative sample and three different algorithms, Rocchio [95], NB [94], and Spy [96], were selected for implementation.

Bayesian classifiers that work explicitly on the possibilities of different assumptions, such as the NB classifier, which is one of the most efficient and most effective algorithms available for certain learning problems, have provided useful practical solutions [97].

The NB classifier can compete with other algorithms and in some cases, it works better than other algorithms [98]. A NB classifier can be considered as a simple Bayesian network, which is used for independence assumptions between features and classes. We chose NB based on the structure and nature of the data, the independent nature of each data source, and the high volume of the data and binary features.

An NB classifier with 4-fold cross validation was used to assess the diagnostic value of every data source. In this assessment, we identified how much of each data source alone was enough to identify the genes of aging (Table 12). The diagnostic value of all data sources was estimated at about 70%, except the Literature. We used the data fusion method to get higher diagnostic value. Because of similar F Measure values, a fusion Kernel of equal weight was selected for each data source.

**Table 12:** Comparison of the evaluation metric across data sources

Data source	Recall	Specificity	Precision	Accuracy	F_Measure
Literature	0.58098	0.61453	0.5888	0.5981	0.58478
Annotation	0.77685	0.78668	0.76645	0.78165	0.77133
Pathways	0.73268	0.74538	0.7204	0.73893	0.72605
Gene Ontology	0.79303	0.78843	0.76315	0.78958	0.77703
Phenotype	0.7946	0.81968	0.8158	0.80695	0.80488
Intrinsic properties	0.67963	0.77035	0.78945	0.71835	0.72965
Sequence	0.6901	0.72828	0.71713	0.70885	0.70305
Interaction	0.7378	0.7724	0.76645	0.7543	0.75135
Gene expression	0.75635	0.82148	0.82235	0.7864	0.78735
Regulatory	0.77355	0.79203	0.77633	0.78163	0.77393

Since our main data did not contain any negative samples, training a model to identify and prioritize new positive genes was based on the three PUL algorithms. An NB classifier was designed following the extraction of a reliable negative sample and positive genes. Genes were assigned positive labels for the final ranking, using the weighting method according to the available data [7] .

The same weight was considered for ranking the candidate genes based on the selected sources. Similarities among the features were weighted in the seed genes and candidate genes, using the following formula, and then sorted based on their total weight:

$$ W(i)=\sum \limits_{i=1}^C\sum \limits_{j=1}^F\left( Candidat{e_{Gene}}_{Feature\left(i,j\right)}\ast \sum \limits_{p=1}^S See{d}_{Gene s\left(p,j\right)}\right), $$

where (*C*) was the number of candidate genes (*n* = 3531), (*F*) was the number of features (*n* = 11, 698), (*S*) was the number of seed genes (*n* = 303) in the problem case, and (*W*) was the weight of each candidate gene.

### Dataset

Aggregate data from 11 human biology databases (Table 13), including 11,698 binary gene features, were collected for 19,462 genes, of which only 303 genes (seed genes) had positive labels for genes involved in aging, derived from the GeneAge database [99].

**Table 13:** Data sources used in Naïve Bayes classifier for candidate aging genes

Data source name	Dataset name	Features detail	Web address
Literature	OBO AgeFactDB	The ageing-related information included both by manual and automatic information extraction from the scientific literature.	https://lov.linkeddata.es/dataset/lov/vocabs/obo http://agefactdb.jenage.de/
Functional annotation	David	The list of all functional annotation.	https://david.ncifcrf.gov/
Biological pathways	Reactome Kegg	The list of biological pathway.	https://reactome.org/ https://www.genome.jp/kegg/pathway.html
Gene Ontology	GO	The Biological Process, Molecular Function, and Cellular Component vocabularies.	http://www.geneontology.org/
Phenotype	HPO OMIM	The list of all ageing-related phenotype and associated gene.	https://hpo.jax.org/ https://www.omim.org/
Intrinsic properties	Pfam PDB	The chromosome number, location, gene segment, gene type, etc.	https://pfam.xfam.org/ https://www.rcsb.org/
Sequence	RefSeq	The list of all known active site, binding site, chain, etc.	https://www.ncbi.nlm.nih.gov/refseq/
Protein-Protein Interaction	HPRD String	The list of each gene had a physical interaction with each of the positive genes.	http://www.hprd.org/ https://string-db.org/
Gene expression	GEO HAGR	The ageing-related expression included tissue type, overexpressed and under expressed, etc.	https://www.ncbi.nlm.nih.gov/geo/ http://genomics.senescence.info/gene_expression/index.php
Regulatory	RegNetwork	The list of all regulatory relationship, such as miRNA, Transcription factor, etc.	http://www.regnetworkweb.org/
Orthologues	CDD HomoloGene OrthoDB	The catalog of orthologous protein-coding genes across vertebrates and known conserved domain.	https://www.ncbi.nlm.nih.gov/Structure/cdd/cdd.shtml https://www.ncbi.nlm.nih.gov/homologene https://www.orthodb.org/

The vector of binary features consisted of 11 main parts, each part of which was equivalent to one of the data sources. The information for each data source was a boolean value, and if any gene contained this value, it scored 1, and otherwise, it scored 0 (Table 2). For example, a part of the biological pathway data contained 330 attributes, which were equivalent to a human pathway in KEGG. If the intended gene was located in this pathway, it scored 1, and otherwise, it scored 0. Also for interaction network data, if each gene had a physical interaction with each of the positive genes, it scored 1, and otherwise, 0. These data were extracted from the String and HPRD databases.

**Fig. 5:**
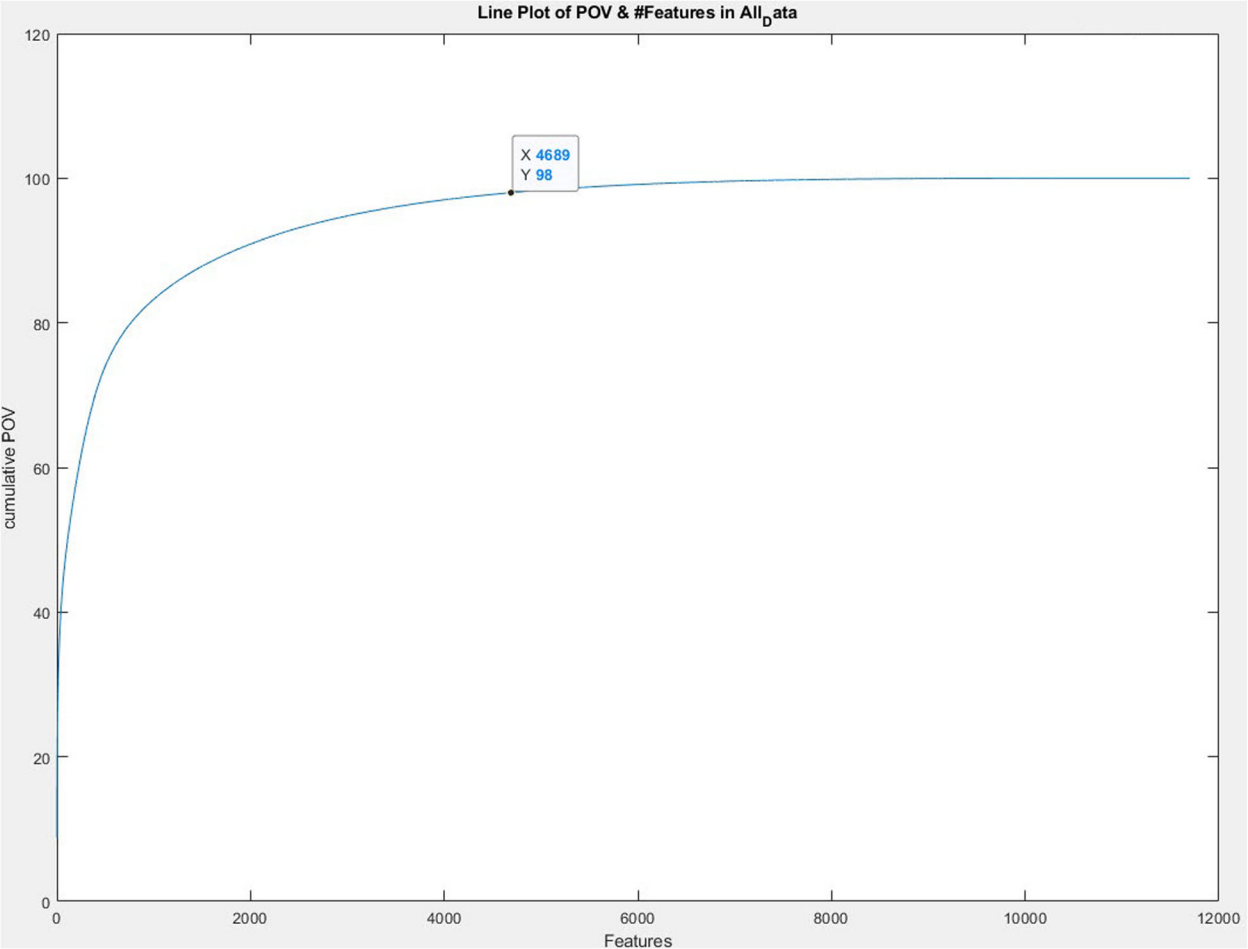
The Percentage of Variance in Principal Component Analysis

Due to the large volume of features, we employed the PCA method to reduce the size of features. Following PCA implementation, our total data set was reduced to 4689 attributes, and the Percentage of Variance (POV) equaled 98% (Fig. 5).

In addition, eight valid data sources from the UCI database (https://archive.ics.uci.edu/ml/index.php) were used to evaluate the efficiency of the algorithms. In each data set, one of the data classes with great sample frequency were unlabeled data. Using algorithms, we identified negative samples and compared them to the original data (Table 3).

## Supplementary information

**Additional file 1:** Comparison of evaluation metric of three algorithms in the UCI databases.

**Additional file 2:** Results of 10-fold cross-validation in the trained and test data.

**Additional file 3:** A list of all huma candidate positive aging genes.

**Additional file 4:** A list of all biological processes associated with the candidate aging genes.

**Additional file 5:** A list of all biological pathways of the candidate genes extracted by FUNRICH.

## Data Availability

Due to the large amount of data, please contact the authors for data requests.

## Abbreviations

GWAS: Genome-Wide Association Study
NB: Naïve Bayes
POV: Percentage of Variance
PPHAGE: Prediction and Prioritization of Human Aging Genes
PUL: Positive-unlabeled learning
ROC: Receiver Operating Characteristic
