# Supplementary material for: Genome-wide prediction and prioritization of human aging genes by data fusion: a machine learning approach
Source: BMC Genomics. 2019 Nov 9;20:832. doi: 10.1186/s12864-019-6140-0 (PMC6842548; doi:10.1186/s12864-019-6140-0)
Supplement: Supplementary file 1 — Additional file 1: Comparison of evaluation metric of three algorithms in the UCI databases. [file 12864_2019_6140_MOESM1_ESM.docx]

Table of distribution sample in the data set 1

| Class percent | number of samples | Class Label |
| --- | --- | --- |
| 74.60 | 564 | Positive |
| 25.40 | 192 | Negative |
| 100 | 756 | Total |

Identification table for test samples in the data set 1

| Number of negative samples | Number of positive samples | Algorithm |
| --- | --- | --- |
| 51 | 175 | NB |
| 46 | 180 | SPY |
| 46 | 180 | Roc-SVM |

Confusion Matrix in the data set 1

| Roc-SVM | | SPY | | NB | |  |
| --- | --- | --- | --- | --- | --- | --- |
| Negative | Positive | Negative | Positive | Negative | Positive |  |
| 27 | 153 | 29 | 151 | 8 | 167 | Positive |
| 43 | 3 | 42 | 4 | 32 | 19 | Negative |

| Comparison of evaluation metric of three algorithms in data set 1 | | | | | | |
| --- | --- | --- | --- | --- | --- | --- |
| F_measure % | Recall % | Precision % | FNR% | FPR% | algorithm |  |
| 92.52 | 89.78 | 95.43 | 4.57 | 37.25 | NB | Parkinson's Disease Classification Data Set |
| 90.15 | 83.89 | 97.42 | 16.11 | 8.70 | SPY |  |
| 91.07 | 85.00 | 98.08 | 15.00 | 6.52 | Roc-SVM |  |

Table of distribution sample in the data set 2

| Class percent | number of samples | Class Label |
| --- | --- | --- |
| 42.03 | 145 | Positive |
| 57.97 | 200 | Negative |
| 100 | 345 | Total |

Identification table for test samples in the data set 2

| Number of negative samples | Number of positive samples | Algorithm |
| --- | --- | --- |
| 68 | 35 | NB |
| 83 | 20 | SPY |
| 83 | 20 | Roc-SVM |

Confusion Matrix in the data set 2

| Roc-SVM | | SPY | | NB | |  |
| --- | --- | --- | --- | --- | --- | --- |
| Negative | Positive | Negative | Positive | Negative | Positive |  |
| 1 | 19 | 0 | 20 | 2 | 33 | Positive |
| 57 | 26 | 53 | 30 | 56 | 12 | Negative |

| Comparison of evaluation metric of three algorithms in data set 2 | | | | | | |
| --- | --- | --- | --- | --- | --- | --- |
| F_measure % | Recall % | Precision % | FNR% | FPR% | algorithm |  |
| 82.50 | 94.29 | 73.33 | 5.71 | 17.65 | NB | Liver Disorders Data Set |
| 57.14 | 100 | 40.00 | 0 | 36.14 | SPY |  |
| 58.46 | 95.00 | 42.22 | 5.00 | 31.33 | Roc-SVM |  |

Table of distribution sample in the data set 3

| Class percent | number of samples | Class Label |
| --- | --- | --- |
| 61.23 | 627 | Positive |
| 38.77 | 397 | Negative |
| 100 | 1024 | Total |

Identification table for test samples in the data set 3

| Number of negative samples | Number of positive samples | Algorithm |
| --- | --- | --- |
| 143 | 164 | NB |
| 126 | 181 | SPY |
| 95 | 212 | Roc-SVM |

Confusion Matrix in the data set 3

| Roc-SVM | | SPY | | NB | |  |
| --- | --- | --- | --- | --- | --- | --- |
| Negative | Positive | Negative | Positive | Negative | Positive |  |
| 35 | 177 | 27 | 154 | 13 | 151 | Positive |
| 89 | 6 | 114 | 12 | 116 | 27 | Negative |

| Comparison of evaluation metric of three algorithms in data set 3 | | | | | | |
| --- | --- | --- | --- | --- | --- | --- |
| F_measure % | Recall % | Precision % | FNR% | FPR% | algorithm |  |
| 88.30 | 92.07 | 84.83 | 7.93 | 18.88 | NB | Cloud Data Set |
| 88.76 | 85.08 | 92.77 | 14.92 | 9.52 | SPY |  |
| 89.62 | 83.49 | 96.72 | 16.51 | 6.32 | Roc-SVM |  |

Table of distribution sample in the data set 4

| Class percent | number of samples | Class Label |
| --- | --- | --- |
| 89.17 | 313 | Positive |
| 10.83 | 38 | Negative |
| 100 | 351 | Total |

Identification table for test samples in the data set 4

| Number of negative samples | Number of positive samples | Algorithm |
| --- | --- | --- |
| 21 | 84 | NB |
| 19 | 86 | SPY |
| 15 | 90 | Roc-SVM |

Confusion Matrix in the data set 4

| Roc-SVM | | SPY | | NB | |  |
| --- | --- | --- | --- | --- | --- | --- |
| Negative | Positive | Negative | Positive | Negative | Positive |  |
| 8 | 82 | 6 | 80 | 7 | 77 | Positive |
| 10 | 5 | 14 | 5 | 11 | 10 | Negative |

| Comparison of evaluation metric of three algorithms in data set 4 | | | | | | |
| --- | --- | --- | --- | --- | --- | --- |
| F_measure % | Recall % | Precision % | FNR% | FPR% | algorithm |  |
| 90.06 | 91.67 | 88.51 | 8.33 | 47.62 | NB | Ionosphere Data Set |
| 93.57 | 93.02 | 94.12 | 6.98 | 26.32 | SPY |  |
| 92.66 | 91.11 | 94.25 | 8.89 | 33.33 | Roc-SVM |  |

Table of distribution sample in the data set 5

| Class percent | number of samples | Class Label |
| --- | --- | --- |
| 13.28 | 332 | Positive |
| 86.72 | 2168 | Negative |
| 100 | 2500 | Total |

Identification table for test samples in the data set 5

| Number of negative samples | Number of positive samples | Algorithm |
| --- | --- | --- |
| 534 | 216 | NB |
| 565 | 185 | SPY |
| 595 | 155 | Roc-SVM |

Confusion Matrix in the data set 5

| Roc-SVM | | SPY | | NB | |  |
| --- | --- | --- | --- | --- | --- | --- |
| Negative | Positive | Negative | Positive | Negative | Positive |  |
| 34 | 121 | 67 | 118 | 96 | 120 | Positive |
| 486 | 109 | 464 | 101 | 478 | 56 | Negative |

| Comparison of evaluation metric of three algorithms in data set 5 | | | | | | |
| --- | --- | --- | --- | --- | --- | --- |
| F_measure % | Recall % | Precision % | FNR% | FPR% | algorithm |  |
| 61.22 | 55.56 | 68.18 | 44.44 | 10.49 | NB | MAGIC Gamma Telescope Data Set |
| 58.42 | 63.78 | 53.88 | 36.22 | 17.88 | SPY |  |
| 62.86 | 78.06 | 52.61 | 21.94 | 18.32 | Roc-SVM |  |

Table of distribution sample in the data set 6

| Class percent | number of samples | Class Label |
| --- | --- | --- |
| 23.80 | 178 | Positive |
| 76.20 | 570 | Negative |
| 100 | 748 | Total |

Identification table for test samples in the data set 6

| Number of negative samples | Number of positive samples | Algorithm |
| --- | --- | --- |
| 138 | 86 | NB |
| 184 | 40 | SPY |
| 154 | 70 | Roc-SVM |

Confusion Matrix in the data set 6

| Roc-SVM | | SPY | | NB | |  |
| --- | --- | --- | --- | --- | --- | --- |
| Negative | Positive | Negative | Positive | Negative | Positive |  |
| 20 | 50 | 4 | 36 | 29 | 57 | Positive |
| 151 | 3 | 162 | 22 | 128 | 10 | Negative |

| Comparison of evaluation metric of three algorithms in data set 6 | | | | | | |
| --- | --- | --- | --- | --- | --- | --- |
| F_measure % | Recall % | Precision % | FNR% | FPR% | algorithm |  |
| 74.51 | 66.28 | 85.07 | 33.72 | 7.25 | NB | Mammographic Mass Data Set |
| 73.47 | 90.00 | 62.07 | 10.00 | 11.96 | SPY |  |
| 81.30 | 71.43 | 94.34 | 28.57 | 1.95 | Roc-SVM |  |

Table of distribution sample in the data set 7

| Class percent | number of samples | Class Label |
| --- | --- | --- |
| 62.74 | 357 | Positive |
| 37.26 | 212 | Negative |
| 100 | 569 | Total |

Identification table for test samples in the data set 7

| Number of negative samples | Number of positive samples | Algorithm |
| --- | --- | --- |
| 65 | 106 | NB |
| 66 | 105 | SPY |
| 40 | 131 | Roc-SVM |

Confusion Matrix in the data set 7

| Roc-SVM | | SPY | | NB | |  |
| --- | --- | --- | --- | --- | --- | --- |
| Negative | Positive | Negative | Positive | Negative | Positive |  |
| 29 | 102 | 11 | 94 | 13 | 93 | Positive |
| 31 | 9 | 60 | 6 | 56 | 9 | Negative |

| Comparison of evaluation metric of three algorithms in data set 7 | | | | | | |
| --- | --- | --- | --- | --- | --- | --- |
| F_measure % | Recall % | Precision % | FNR% | FPR% | algorithm |  |
| 89.42 | 87.74 | 91.18 | 12.26 | 13.85 | NB | Breast Cancer Wisconsin (Diagnostic) Data Set |
| 91.71 | 89.52 | 94.00 | 10.48 | 9.09 | SPY |  |
| 84.30 | 77.86 | 91.89 | 22.14 | 22.50 | Roc-SVM |  |

Table of distribution sample in the data set 8

| Class percent | number of samples | Class Label |
| --- | --- | --- |
| 46.63 | 97 | Positive |
| 53.37 | 111 | Negative |
| 100 | 208 | Total |

Identification table for test samples in the data set 8

| Number of negative samples | Number of positive samples | Algorithm |
| --- | --- | --- |
| 37 | 25 | NB |
| 36 | 26 | SPY |
| 30 | 32 | Roc-SVM |

Confusion Matrix in the data set 8

| Roc-SVM | | SPY | | NB | |  |
| --- | --- | --- | --- | --- | --- | --- |
| Negative | Positive | Negative | Positive | Negative | Positive |  |
| 5 | 27 | 2 | 24 | 1 | 24 | Positive |
| 27 | 3 | 30 | 6 | 33 | 4 | Negative |

| Comparison of evaluation metric of three algorithms in data set 8 | | | | | | |
| --- | --- | --- | --- | --- | --- | --- |
| F_measure % | Recall % | Precision % | FNR% | FPR% | algorithm |  |
| 89.42 | 87.74 | 91.18 | 12.26 | 13.85 | NB | Connectionist Bench (Sonar, Mines vs. Rocks) Data Set |
| 85.71 | 92.31 | 80.00 | 7.69 | 16.67 | SPY |  |
| 84.30 | 77.86 | 91.89 | 22.14 | 22.50 | Roc-SVM |  |
