# Supplementary material for: Genome-wide prediction and prioritization of human aging genes by data fusion: a machine learning approach
Source: BMC Genomics. 2019 Nov 9;20:832. doi: 10.1186/s12864-019-6140-0 (PMC6842548; doi:10.1186/s12864-019-6140-0)
Supplement: Supplementary file 2 — Additional file 2: Results of 10-fold cross-validation in the trained and test data. [file 12864_2019_6140_MOESM2_ESM.docx]

Confusion matrix for training data

| Fold10 | | Fold9 | | Fold8 | | Fold7 | | Fold6 | | Fold5 | | Fold4 | | Fold3 | | Fold2 | | Fold1 | |  |
| --- | --- | --- | --- | --- | --- | --- | --- | --- | --- | --- | --- | --- | --- | --- | --- | --- | --- | --- | --- | --- |
| - | + | - | + | - | + | - | + | - | + | - | + | - | + | - | + | - | + | - | + |  |
| 69 | 227 | 67 | 228 | 68 | 227 | 70 | 225 | 69 | 226 | 64 | 231 | 63 | 232 | 68 | 227 | 65 | 230 | 68 | 228 | + |
| 215 | 57 | 217 | 56 | 215 | 58 | 212 | 61 | 215 | 58 | 219 | 54 | 216 | 57 | 219 | 54 | 213 | 59 | 211 | 61 | - |

Performance evaluation for training data

| AUC % | Accuracy % | F_measure % | Recall % | Precision % | Fold# |
| --- | --- | --- | --- | --- | --- |
| 82.38 | 77.29 | 77.94 | 77.02 | 78.90 | 1 |
| 82.93 | 78.13 | 78.76 | 77.97 | 79.59 | 2 |
| 83.81 | 78.52 | 78.81 | 76.95 | 80.78 | 3 |
| 84.00 | 78.87 | 79.45 | 78.64 | 80.28 | 4 |
| 83.61 | 79.22 | 79.65 | 78.30 | 81.05 | 5 |
| 83.08 | 77.64 | 78.06 | 76.61 | 79.58 | 6 |
| 82.57 | 76.94 | 77.45 | 76.27 | 78.67 | 7 |
| 82.84 | 77.81 | 78.28 | 76.95 | 79.65 | 8 |
| 83.28 | 78.34 | 78.76 | 77.29 | 80.28 | 9 |
| 83.27 | 77.82 | 78.27 | 76.69 | 79.93 | 10 |

| 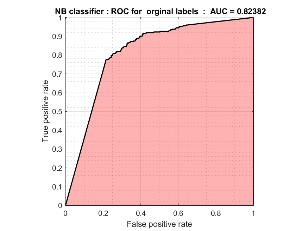 | 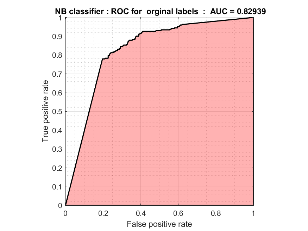 | 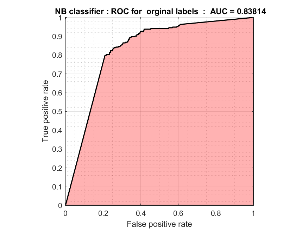 | 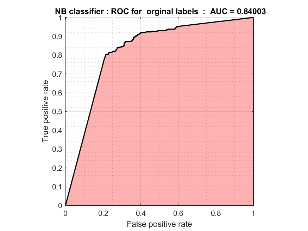 | 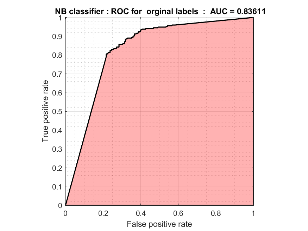 |
| --- | --- | --- | --- | --- |
| 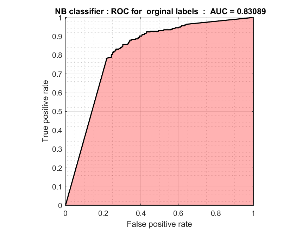 | 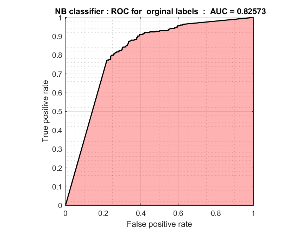 | 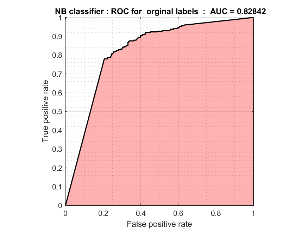 | 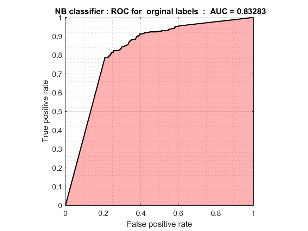 | 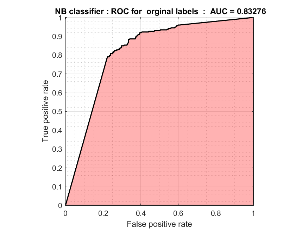 |

Confusion matrix for test data

| Fold10 | | Fold9 | | Fold8 | | Fold7 | | Fold6 | | Fold5 | | Fold4 | | Fold3 | | Fold2 | | Fold1 | |  |
| --- | --- | --- | --- | --- | --- | --- | --- | --- | --- | --- | --- | --- | --- | --- | --- | --- | --- | --- | --- | --- |
| - | + | - | + | - | + | - | + | - | + | - | + | - | + | - | + | - | + | - | + |  |
| 7 | 25 | 6 | 27 | 6 | 27 | 6 | 27 | 4 | 29 | 12 | 21 | 15 | 18 | 7 | 26 | 9 | 24 | 6 | 26 | + |
| 23 | 8 | 22 | 8 | 24 | 6 | 26 | 4 | 23 | 7 | 22 | 8 | 26 | 4 | 19 | 11 | 25 | 6 | 27 | 4 | - |

Performance evaluation for test data

| AUC % | Accuracy % | F_measure % | Recall % | Precision % | Fold# |
| --- | --- | --- | --- | --- | --- |
| 90.97 | 84.13 | 83.87 | 81.25 | 86.67 | 1 |
| 84.31 | 76.56 | 76.19 | 72.72 | 80.00 | 2 |
| 75.30 | 71.43 | 74.29 | 78.79 | 70.27 | 3 |
| 72.52 | 69.84 | 65.45 | 54.54 | 81.81 | 4 |
| 74.59 | 68.25 | 67.74 | 63.64 | 72.41 | 5 |
| 83.83 | 82.53 | 84.05 | 87.88 | 80.55 | 6 |
| 88.99 | 84.13 | 84.37 | 81.82 | 87.09 | 7 |
| 83.73 | 80.95 | 81.82 | 81.82 | 81.82 | 8 |
| 80.70 | 77.78 | 79.41 | 81.82 | 77.14 | 9 |
| 78.83 | 76.19 | 76.92 | 78.12 | 75.76 | 10 |

| 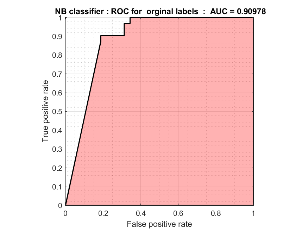 | 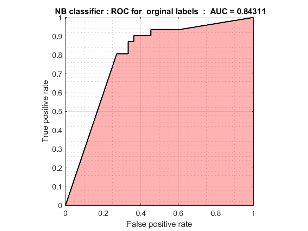 | 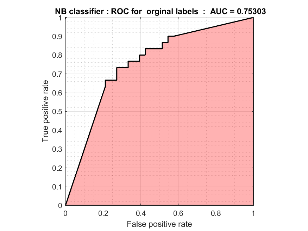 | 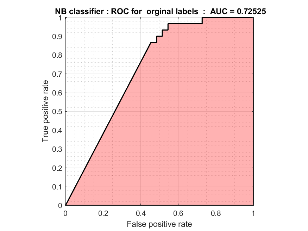 | 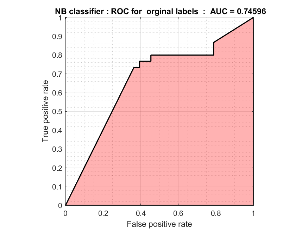 |
| --- | --- | --- | --- | --- |
| 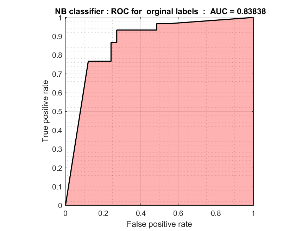 | 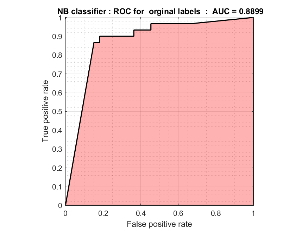 | 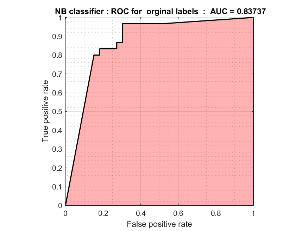 | 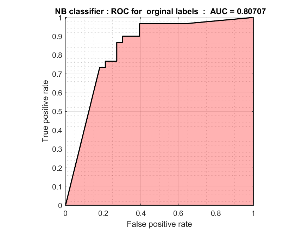 | 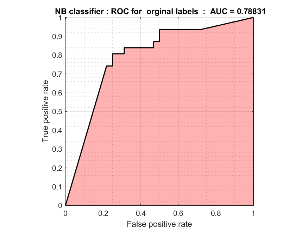 |
