# Supplementary material for: Genome-wide prediction and prioritization of human aging genes by data fusion: a machine learning approach
Source: BMC Genomics. 2019 Nov 9;20:832. doi: 10.1186/s12864-019-6140-0 (PMC6842548; doi:10.1186/s12864-019-6140-0)
Supplement: Supplementary file 3 — Additional file 3: A list of all huma candidate positive aging genes. [file 12864_2019_6140_MOESM3_ESM.docx]

| 1 | NAP1L4 |
| --- | --- |
| 2 | CCNI |
| 3 | RPL3 |
| 4 | FZD5 |
| 5 | BRD2 |
| 6 | ATP8A2 |
| 7 | SRSF11 |
| 8 | BBIP1 |
| 9 | IL10 |
| 10 | FYCO1 |
| 11 | PSMB2 |
| 12 | NSF |
| 13 | OAZ1 |
| 14 | ZFP36L1 |
| 15 | PCLO |
| 16 | GAB2 |
| 17 | QKI |
| 18 | ZNF638 |
| 19 | RGS3 |
| 20 | XPO6 |
| 21 | ATP8B1 |
| 22 | ITM2C |
| 23 | RBFOX1 |
| 24 | DLC1 |
| 25 | MVK |
| 26 | DEK |
| 27 | BCAR1 |
| 28 | SNX5 |
| 29 | FSCN1 |
| 30 | EDC3 |
| 31 | ATP5A1 |
| 32 | SIGMAR1 |
| 33 | PPP2CA |
| 34 | RYR2 |
| 35 | TM9SF3 |
| 36 | FKBP1A |
| 37 | AK3 |
| 38 | BCAS3 |
| 39 | RBCK1 |
| 40 | PLCB1 |
| 41 | HSP90B1 |
| 42 | RCC1 |
| 43 | VPS26A |
| 44 | HSPG2 |
| 45 | NFATC1 |
| 46 | SPRED2 |
| 47 | ERBB3 |
| 48 | ZDHHC17 |
| 49 | MCM3 |
| 50 | SLC16A2 |
| 51 | PDCD6 |
| 52 | TUFM |
| 53 | SLC3A1 |
| 54 | POLR1C |
| 55 | RCAN1 |
| 56 | KANK1 |
| 57 | PEA15 |
| 58 | TLR3 |
| 59 | PDE4DIP |
| 60 | IKBKG |
| 61 | PLEC |
| 62 | ERAL1 |
| 63 | CEACAM1 |
| 64 | RB1CC1 |
| 65 | MRPS27 |
| 66 | KALRN |
| 67 | NDUFB9 |
| 68 | CSMD1 |
| 69 | DNMT1 |
| 70 | SSX2IP |
| 71 | MFF |
| 72 | COBL |
| 73 | PLAGL1 |
| 74 | TWF1 |
| 75 | RSL1D1 |
| 76 | LRP5 |
| 77 | ETFA |
| 78 | ATP1B3 |
| 79 | FADS1 |
| 80 | DST |
| 81 | KRT8 |
| 82 | CHRNB2 |
| 83 | GLE1 |
| 84 | PA2G4 |
| 85 | PIH1D1 |
| 86 | STRA6 |
| 87 | PITPNA |
| 88 | KPNA3 |
| 89 | PTPRJ |
| 90 | USP15 |
| 91 | XPO7 |
| 92 | RORA |
| 93 | PDXDC1 |
| 94 | PTGES2 |
| 95 | USO1 |
| 96 | SNX1 |
| 97 | PKD1 |
| 98 | PNMT |
| 99 | LLGL2 |
| 100 | ASH2L |
| 101 | SEC24B |
| 102 | TM4SF1 |
| 103 | TNKS |
| 104 | EIF3E |
| 105 | WWOX |
| 106 | ZAP70 |
| 107 | AUTS2 |
| 108 | RPL27A |
| 109 | RBBP7 |
| 110 | MAFB |
| 111 | VPS37B |
| 112 | RPL23 |
| 113 | HNRNPUL1 |
| 114 | HNRNPU |
| 115 | SCRIB |
| 116 | PKD2 |
| 117 | TAB2 |
| 118 | LTBR |
| 119 | STT3A |
| 120 | SYNE2 |
| 121 | ILF3 |
| 122 | AES |
| 123 | DOCK2 |
| 124 | DUSP6 |
| 125 | SRP68 |
| 126 | PUM1 |
| 127 | SNRPC |
| 128 | DDX20 |
| 129 | PELP1 |
| 130 | UBR4 |
| 131 | PDE4D |
| 132 | GNAS |
| 133 | USP7 |
| 134 | MYLK |
| 135 | LIG4 |
| 136 | RABEP1 |
| 137 | IRF7 |
| 138 | DMPK |
| 139 | DOCK5 |
| 140 | RAD21 |
| 141 | RBBP8 |
| 142 | CLPB |
| 143 | TMBIM6 |
| 144 | ARID1A |
| 145 | PFN2 |
| 146 | NFATC4 |
| 147 | TCF7L2 |
| 148 | STK3 |
| 149 | ZNF410 |
| 150 | WDR81 |
| 151 | PDE2A |
| 152 | RPL7A |
| 153 | MAGI2 |
| 154 | AKAP8L |
| 155 | FLNA |
| 156 | PDE4B |
| 157 | PTPRN2 |
| 158 | SEC13 |
| 159 | ANXA3 |
| 160 | RAD54L |
| 161 | ST5 |
| 162 | CEP164 |
| 163 | BZW2 |
| 164 | ATG9A |
| 165 | FBXW11 |
| 166 | WT1 |
| 167 | EEF1D |
| 168 | TBC1D5 |
| 169 | APPL2 |
| 170 | FAN1 |
| 171 | CACNA1C |
| 172 | DDHD2 |
| 173 | FMN2 |
| 174 | SATB1 |
| 175 | SCFD1 |
| 176 | ATP6V0A2 |
| 177 | TOR1AIP1 |
| 178 | FLNB |
| 179 | ENOSF1 |
| 180 | PLOD2 |
| 181 | RNF130 |
| 182 | ARID1B |
| 183 | SNX12 |
| 184 | FXR1 |
| 185 | SLC25A6 |
| 186 | HNRNPD |
| 187 | TNXB |
| 188 | ERBB4 |
| 189 | PFN1 |
| 190 | DDX5 |
| 191 | SPAG5 |
| 192 | HEXB |
| 193 | HNF1A |
| 194 | CD74 |
| 195 | PAX6 |
| 196 | ANG |
| 197 | GOSR2 |
| 198 | GUK1 |
| 199 | ASNS |
| 200 | PIM1 |
| 201 | IDI1 |
| 202 | SNRPA |
| 203 | BAIAP2 |
| 204 | ANXA2 |
| 205 | CELF2 |
| 206 | NOMO2 |
| 207 | CLK3 |
| 208 | RRAS2 |
| 209 | GBA |
| 210 | ANK3 |
| 211 | KTN1 |
| 212 | RFC5 |
| 213 | ITCH |
| 214 | LONP1 |
| 215 | NDRG4 |
| 216 | GSTT1 |
| 217 | CYFIP1 |
| 218 | CHRFAM7A |
| 219 | SFRP1 |
| 220 | ITGA8 |
| 221 | TPI1 |
| 222 | HMG20A |
| 223 | KIAA0319L |
| 224 | SGK1 |
| 225 | RACGAP1 |
| 226 | CACNB2 |
| 227 | ITGAV |
| 228 | CXCR4 |
| 229 | HGS |
| 230 | RPS15 |
| 231 | GPM6A |
| 232 | SEC16A |
| 233 | EPHB1 |
| 234 | APLP1 |
| 235 | ASXL1 |
| 236 | COL7A1 |
| 237 | CDK5RAP3 |
| 238 | PDHA1 |
| 239 | DIAPH1 |
| 240 | BMP4 |
| 241 | FAM133B |
| 242 | MTHFD1 |
| 243 | MED15 |
| 244 | CDH2 |
| 245 | NFKBIE |
| 246 | EHD1 |
| 247 | VAMP2 |
| 248 | NVL |
| 249 | PDIA6 |
| 250 | SOS1 |
| 251 | CD47 |
| 252 | SLC11A2 |
| 253 | SERPINA1 |
| 254 | TUBGCP2 |
| 255 | EIF4E |
| 256 | PSMA4 |
| 257 | RAD51C |
| 258 | TLR2 |
| 259 | FAM120A |
| 260 | MDH2 |
| 261 | TICAM1 |
| 262 | PDHX |
| 263 | CAPNS1 |
| 264 | CYP26B1 |
| 265 | CTNND1 |
| 266 | BBS1 |
| 267 | COG5 |
| 268 | DBNL |
| 269 | SNRPD2 |
| 270 | ARAP1 |
| 271 | MANBA |
| 272 | EID1 |
| 273 | CACNA1D |
| 274 | PID1 |
| 275 | TYK2 |
| 276 | OXA1L |
| 277 | LILRB1 |
| 278 | CASP8 |
| 279 | CLN3 |
| 280 | ECE1 |
| 281 | OLA1 |
| 282 | HMOX2 |
| 283 | CIZ1 |
| 284 | CDH11 |
| 285 | AKAP13 |
| 286 | GRIP1 |
| 287 | UIMC1 |
| 288 | NOMO3 |
| 289 | STXBP1 |
| 290 | THOC5 |
| 291 | WDR60 |
| 292 | LAMA5 |
| 293 | POR |
| 294 | CDIPT |
| 295 | PSMC1 |
| 296 | SMC4 |
| 297 | NDUFA10 |
| 298 | KCNMA1 |
| 299 | GBA2 |
| 300 | MEIS1 |
| 301 | LIMA1 |
| 302 | PTPN2 |
| 303 | GET4 |
| 304 | COL6A1 |
| 305 | HEG1 |
| 306 | HNF4A |
| 307 | PRKCZ |
| 308 | CLCN3 |
| 309 | MAZ |
| 310 | MACF1 |
| 311 | MAP4 |
| 312 | FEM1B |
| 313 | SET |
| 314 | PANK2 |
| 315 | PTPRM |
| 316 | CD81 |
| 317 | SETD2 |
| 318 | CKAP4 |
| 319 | POMT1 |
| 320 | IFT122 |
| 321 | MASP1 |
| 322 | PSMC3IP |
| 323 | PSEN2 |
| 324 | FZD1 |
| 325 | PRKCH |
| 326 | SNX19 |
| 327 | OGT |
| 328 | IKZF3 |
| 329 | FKTN |
| 330 | SRPK2 |
| 331 | PTH1R |
| 332 | HSPA2 |
| 333 | AP1G1 |
| 334 | LUC7L |
| 335 | MCM2 |
| 336 | PIP4K2A |
| 337 | IDUA |
| 338 | DBI |
| 339 | SMYD3 |
| 340 | UBAC2 |
| 341 | MPV17 |
| 342 | QARS |
| 343 | STIM1 |
| 344 | HBA2 |
| 345 | NANS |
| 346 | SEC23A |
| 347 | PRPS1 |
| 348 | XPOT |
| 349 | RPL27 |
| 350 | RPL29 |
| 351 | WNT7B |
| 352 | ARHGAP32 |
| 353 | VAC14 |
| 354 | TNS3 |
| 355 | AP2S1 |
| 356 | SLU7 |
| 357 | NKTR |
| 358 | SCARB2 |
| 359 | UBE2E2 |
| 360 | RAPGEF2 |
| 361 | CCT8 |
| 362 | USP11 |
| 363 | DMD |
| 364 | SERPINE2 |
| 365 | SNAP29 |
| 366 | DHX36 |
| 367 | PRCP |
| 368 | GJA5 |
| 369 | DERL1 |
| 370 | NMT1 |
| 371 | SIK1 |
| 372 | PLAT |
| 373 | TET2 |
| 374 | GATAD2B |
| 375 | LPIN2 |
| 376 | MCCC1 |
| 377 | PPP1CB |
| 378 | TARDBP |
| 379 | RPA3 |
| 380 | TCF12 |
| 381 | TNS1 |
| 382 | TRRAP |
| 383 | TCF4 |
| 384 | NACA |
| 385 | NPM1 |
| 386 | DARS |
| 387 | GGA1 |
| 388 | PLAUR |
| 389 | GATA6 |
| 390 | ANLN |
| 391 | CITED1 |
| 392 | STMN1 |
| 393 | ROBO1 |
| 394 | CHRNA7 |
| 395 | N4BP2L2 |
| 396 | PRMT1 |
| 397 | MCM4 |
| 398 | SRPK1 |
| 399 | GAB1 |
| 400 | COG4 |
| 401 | TCTN1 |
| 402 | TIAM1 |
| 403 | NUDT21 |
| 404 | IQCB1 |
| 405 | PON2 |
| 406 | CSK |
| 407 | EIF3K |
| 408 | ATG3 |
| 409 | CRH |
| 410 | PTPRC |
| 411 | SERPINF1 |
| 412 | TPD52 |
| 413 | BMPR1B |
| 414 | DYNC1I2 |
| 415 | HP1BP3 |
| 416 | TARBP2 |
| 417 | NTRK3 |
| 418 | GATAD2A |
| 419 | ZMYM4 |
| 420 | EIF4E2 |
| 421 | VAT1 |
| 422 | AGR2 |
| 423 | NUDC |
| 424 | PCK2 |
| 425 | RPS2 |
| 426 | ASS1 |
| 427 | UNC13B |
| 428 | LPIN1 |
| 429 | GALNT1 |
| 430 | CS |
| 431 | CNOT2 |
| 432 | PHKG2 |
| 433 | NAP1L1 |
| 434 | BAG6 |
| 435 | NAGK |
| 436 | SPARCL1 |
| 437 | PDLIM7 |
| 438 | UBC |
| 439 | EPB41L3 |
| 440 | WWP2 |
| 441 | TCF25 |
| 442 | ATP5G2 |
| 443 | ITGB1 |
| 444 | JUP |
| 445 | ARAF |
| 446 | SMG1 |
| 447 | ARNT2 |
| 448 | CPD |
| 449 | PDK2 |
| 450 | GAS7 |
| 451 | RALY |
| 452 | REV3L |
| 453 | ZNF207 |
| 454 | RAPGEF6 |
| 455 | PSMA1 |
| 456 | ZP3 |
| 457 | ARPC4 |
| 458 | ZYX |
| 459 | CYFIP2 |
| 460 | EPHX1 |
| 461 | ITGB2 |
| 462 | DYNC1I1 |
| 463 | ZNF592 |
| 464 | CLCN7 |
| 465 | NARS |
| 466 | GCC2 |
| 467 | BPGM |
| 468 | GDI2 |
| 469 | PAK4 |
| 470 | PAPLN |
| 471 | CHMP2B |
| 472 | RPL30 |
| 473 | EHMT1 |
| 474 | HDAC6 |
| 475 | EBP |
| 476 | PTDSS1 |
| 477 | ADSL |
| 478 | S100A8 |
| 479 | MERTK |
| 480 | COL18A1 |
| 481 | DYRK1A |
| 482 | SMAD4 |
| 483 | KIAA0586 |
| 484 | CX3CR1 |
| 485 | STAT1 |
| 486 | FDPS |
| 487 | AP3M1 |
| 488 | CTCF |
| 489 | TEX10 |
| 490 | PHF1 |
| 491 | GFAP |
| 492 | FKBP4 |
| 493 | AGFG1 |
| 494 | ANGPT1 |
| 495 | SLC29A1 |
| 496 | EPB41 |
| 497 | HDAC9 |
| 498 | LPP |
| 499 | FANCC |
| 500 | ESD |
| 501 | F13A1 |
| 502 | RPS3A |
| 503 | SPRY1 |
| 504 | ALAD |
| 505 | PPARD |
| 506 | SKIL |
| 507 | CLIC1 |
| 508 | G3BP2 |
| 509 | RNF8 |
| 510 | SORD |
| 511 | ALDH1A1 |
| 512 | PGK1 |
| 513 | TGIF1 |
| 514 | ARHGEF2 |
| 515 | CEP170 |
| 516 | RBPJ |
| 517 | RRBP1 |
| 518 | EIF2S1 |
| 519 | PSPC1 |
| 520 | TGFBI |
| 521 | HDAC7 |
| 522 | GAPDH |
| 523 | NPTN |
| 524 | RAD50 |
| 525 | ROCK2 |
| 526 | LTBP3 |
| 527 | MDFI |
| 528 | TXN2 |
| 529 | SSR2 |
| 530 | KITLG |
| 531 | SIRT2 |
| 532 | CHORDC1 |
| 533 | NDRG2 |
| 534 | GABARAPL1 |
| 535 | RANBP3 |
| 536 | ITPK1 |
| 537 | EIF3CL |
| 538 | RNPS1 |
| 539 | DDAH2 |
| 540 | GREM1 |
| 541 | NCK1 |
| 542 | SRRM1 |
| 543 | PPM1A |
| 544 | NEDD4L |
| 545 | HMGCR |
| 546 | MYH14 |
| 547 | AMOTL2 |
| 548 | SCYL1 |
| 549 | PIAS3 |
| 550 | RBBP6 |
| 551 | TAGLN2 |
| 552 | SLC39A13 |
| 553 | MTHFR |
| 554 | DAXX |
| 555 | SWAP70 |
| 556 | ARCN1 |
| 557 | BLMH |
| 558 | PPP2R1A |
| 559 | HBA1 |
| 560 | BEST1 |
| 561 | MSH6 |
| 562 | WDR11 |
| 563 | RHOA |
| 564 | IRF5 |
| 565 | DHX30 |
| 566 | DCTN1 |
| 567 | MAPK13 |
| 568 | INPP5F |
| 569 | POLR3E |
| 570 | CTSD |
| 571 | RPS6 |
| 572 | SMAD7 |
| 573 | DUT |
| 574 | NEDD9 |
| 575 | PIK3R3 |
| 576 | NF1 |
| 577 | NUTF2 |
| 578 | CAMKK2 |
| 579 | SEC61A1 |
| 580 | GM2A |
| 581 | RPL21 |
| 582 | SYT1 |
| 583 | BNIP3L |
| 584 | XRCC1 |
| 585 | NOP2 |
| 586 | RNH1 |
| 587 | ANXA11 |
| 588 | SPG21 |
| 589 | MICAL1 |
| 590 | PSME3 |
| 591 | BCOR |
| 592 | YES1 |
| 593 | DCTD |
| 594 | CNP |
| 595 | SNAP23 |
| 596 | CSNK2A1 |
| 597 | EPB41L2 |
| 598 | PHF21A |
| 599 | RAB27A |
| 600 | XPO1 |
| 601 | PDPN |
| 602 | HMG20B |
| 603 | ITGB4 |
| 604 | VPS45 |
| 605 | ENO1 |
| 606 | SUPT6H |
| 607 | TNIP1 |
| 608 | PCNP |
| 609 | ANK2 |
| 610 | GTF2I |
| 611 | HHEX |
| 612 | PARD3 |
| 613 | EZH2 |
| 614 | BRAF |
| 615 | RHOC |
| 616 | RPN2 |
| 617 | BBX |
| 618 | ATP2C1 |
| 619 | TTC3 |
| 620 | PLD2 |
| 621 | RNF34 |
| 622 | EIF4A1 |
| 623 | SEMA3C |
| 624 | SUN2 |
| 625 | FHL1 |
| 626 | ANXA7 |
| 627 | BACH2 |
| 628 | CCDC88A |
| 629 | CSNK1A1 |
| 630 | TJP1 |
| 631 | PARN |
| 632 | BCS1L |
| 633 | IFT80 |
| 634 | AURKA |
| 635 | DIABLO |
| 636 | SLIT2 |
| 637 | DAP3 |
| 638 | PSME1 |
| 639 | WDR48 |
| 640 | FABP5 |
| 641 | RAP1A |
| 642 | KCNAB2 |
| 643 | TACC2 |
| 644 | SULF2 |
| 645 | ADNP |
| 646 | SUOX |
| 647 | ATG7 |
| 648 | RPS20 |
| 649 | EZR |
| 650 | RAN |
| 651 | TPP1 |
| 652 | RBM17 |
| 653 | EIF3H |
| 654 | RPLP0 |
| 655 | TUBB3 |
| 656 | RNF13 |
| 657 | JAK3 |
| 658 | FKBP10 |
| 659 | FLCN |
| 660 | GPBP1 |
| 661 | BCL6 |
| 662 | ATXN3 |
| 663 | CD86 |
| 664 | RPS19 |
| 665 | HNRNPC |
| 666 | GIT1 |
| 667 | SORL1 |
| 668 | TRIOBP |
| 669 | PGD |
| 670 | CASP7 |
| 671 | TRAF6 |
| 672 | APOD |
| 673 | ELP3 |
| 674 | UBE2D3 |
| 675 | SRRM2 |
| 676 | GFM1 |
| 677 | CCND3 |
| 678 | SLC26A2 |
| 679 | CTBP2 |
| 680 | DGUOK |
| 681 | TAX1BP1 |
| 682 | CAPN1 |
| 683 | TSC22D1 |
| 684 | COL6A3 |
| 685 | ATP6V1D |
| 686 | RPL10 |
| 687 | ENG |
| 688 | TBC1D1 |
| 689 | COPS5 |
| 690 | SF1 |
| 691 | CSNK2B |
| 692 | SAR1A |
| 693 | PACSIN2 |
| 694 | SASH1 |
| 695 | FAU |
| 696 | SHMT2 |
| 697 | CEP120 |
| 698 | EIF2AK2 |
| 699 | FKRP |
| 700 | FARP1 |
| 701 | CPT1A |
| 702 | RPL15 |
| 703 | TFG |
| 704 | SEC31A |
| 705 | HLA-C |
| 706 | RBMS1 |
| 707 | CDK5RAP1 |
| 708 | BSG |
| 709 | BDH1 |
| 710 | CDC6 |
| 711 | PAICS |
| 712 | BIRC2 |
| 713 | AGER |
| 714 | UTRN |
| 715 | SPATS2L |
| 716 | TTN |
| 717 | PRPF31 |
| 718 | TCEA2 |
| 719 | CTDP1 |
| 720 | PDX1 |
| 721 | FXN |
| 722 | DNM1L |
| 723 | GABARAPL2 |
| 724 | MBNL1 |
| 725 | SNX10 |
| 726 | RAI14 |
| 727 | SLC3A2 |
| 728 | MTMR2 |
| 729 | METAP2 |
| 730 | MAD2L2 |
| 731 | ARPC1B |
| 732 | PFKM |
| 733 | COL4A2 |
| 734 | TBXAS1 |
| 735 | DGKI |
| 736 | RNF40 |
| 737 | MME |
| 738 | PLD3 |
| 739 | BANP |
| 740 | CAPRIN1 |
| 741 | PSMA5 |
| 742 | RBM39 |
| 743 | CHL1 |
| 744 | TBCD |
| 745 | LGMN |
| 746 | RPS5 |
| 747 | TBL1XR1 |
| 748 | RUSC1 |
| 749 | CTNNA1 |
| 750 | ANXA1 |
| 751 | INPP5K |
| 752 | NCKAP1L |
| 753 | RPS3 |
| 754 | PALLD |
| 755 | PHF20 |
| 756 | LZTS2 |
| 757 | TWF2 |
| 758 | MYO10 |
| 759 | MKLN1 |
| 760 | CCNK |
| 761 | SYMPK |
| 762 | TBC1D9B |
| 763 | CYP19A1 |
| 764 | PSMA6 |
| 765 | UBA52 |
| 766 | NDEL1 |
| 767 | PRMT5 |
| 768 | STX4 |
| 769 | PICALM |
| 770 | BAD |
| 771 | TPM3 |
| 772 | CCNY |
| 773 | HERPUD1 |
| 774 | NQO1 |
| 775 | LPAR1 |
| 776 | SYNE1 |
| 777 | ENC1 |
| 778 | NUP62 |
| 779 | EIF2B3 |
| 780 | PHB |
| 781 | ARSA |
| 782 | IRF3 |
| 783 | PI4KA |
| 784 | PNKP |
| 785 | CIAPIN1 |
| 786 | AGPAT1 |
| 787 | BRD4 |
| 788 | CDK4 |
| 789 | EIF4G2 |
| 790 | SRP14 |
| 791 | EPHX2 |
| 792 | NOX4 |
| 793 | TRIM2 |
| 794 | FKBP8 |
| 795 | GLB1 |
| 796 | EIF5A |
| 797 | CPNE3 |
| 798 | MAPRE2 |
| 799 | ATP13A2 |
| 800 | AHI1 |
| 801 | XIAP |
| 802 | DHCR7 |
| 803 | PPP6R3 |
| 804 | SMARCAL1 |
| 805 | CPE |
| 806 | SHISA5 |
| 807 | WBP1 |
| 808 | H6PD |
| 809 | DNAJB1 |
| 810 | DCAF11 |
| 811 | OPA1 |
| 812 | DDX11 |
| 813 | PKP4 |
| 814 | ATG4B |
| 815 | PIGN |
| 816 | CDKN1C |
| 817 | AKT2 |
| 818 | ATP6V1A |
| 819 | PTPN13 |
| 820 | NDUFV1 |
| 821 | GPRC5B |
| 822 | WWTR1 |
| 823 | RPSA |
| 824 | WARS |
| 825 | RPL13 |
| 826 | FGR |
| 827 | TMSB4X |
| 828 | COPS6 |
| 829 | COX4I1 |
| 830 | AMFR |
| 831 | FTO |
| 832 | RAP1B |
| 833 | HGF |
| 834 | IFI27 |
| 835 | PSMD4 |
| 836 | CTNND2 |
| 837 | NPRL3 |
| 838 | GOLGA2 |
| 839 | TF |
| 840 | GTF3C5 |
| 841 | RPL9 |
| 842 | TCP1 |
| 843 | LRP6 |
| 844 | TMEM59 |
| 845 | IL4R |
| 846 | CBLB |
| 847 | FLII |
| 848 | GEMIN4 |
| 849 | FGFR4 |
| 850 | CCBE1 |
| 851 | RPS10 |
| 852 | TIAL1 |
| 853 | RPL7 |
| 854 | MLST8 |
| 855 | CD34 |
| 856 | CD36 |
| 857 | SPINT2 |
| 858 | SLMAP |
| 859 | TMEM67 |
| 860 | CD9 |
| 861 | APBB2 |
| 862 | MAVS |
| 863 | IFITM1 |
| 864 | SEMA3A |
| 865 | FBLN1 |
| 866 | GRB10 |
| 867 | PROX1 |
| 868 | EIF3C |
| 869 | PLK1 |
| 870 | IQGAP1 |
| 871 | FERMT1 |
| 872 | H3F3B |
| 873 | SIL1 |
| 874 | FLOT1 |
| 875 | CAP1 |
| 876 | PRKAG1 |
| 877 | UBN1 |
| 878 | SREBF1 |
| 879 | XPNPEP1 |
| 880 | SYT12 |
| 881 | SH3BP4 |
| 882 | EEF1B2 |
| 883 | PAK1 |
| 884 | TFIP11 |
| 885 | OXR1 |
| 886 | KATNB1 |
| 887 | MAEA |
| 888 | COPB1 |
| 889 | PSMC5 |
| 890 | TUBA4A |
| 891 | TCIRG1 |
| 892 | KIAA1217 |
| 893 | FGFRL1 |
| 894 | GLUL |
| 895 | CYP1B1 |
| 896 | CD59 |
| 897 | LMO7 |
| 898 | SART3 |
| 899 | GRIN2A |
| 900 | OTUB1 |
| 901 | TUBA1A |
| 902 | FLG |
| 903 | C1S |
| 904 | RSU1 |
| 905 | MEIS2 |
| 906 | MAP2K5 |
| 907 | CBX5 |
| 908 | IKBKE |
| 909 | SH3BP2 |
| 910 | CAPN2 |
| 911 | FARSA |
| 912 | IQGAP2 |
| 913 | PRKCB |
| 914 | SLC16A1 |
| 915 | IFITM3 |
| 916 | WBP2 |
| 917 | TMEM30A |
| 918 | GPC1 |
| 919 | PPP3R1 |
| 920 | CASP3 |
| 921 | HBS1L |
| 922 | CAPN3 |
| 923 | PAX8 |
| 924 | TBC1D22A |
| 925 | MAP4K4 |
| 926 | BAG5 |
| 927 | RPL22 |
| 928 | GNA13 |
| 929 | CKAP5 |
| 930 | CORO1C |
| 931 | MARK2 |
| 932 | VPS13A |
| 933 | CAND1 |
| 934 | RBM23 |
| 935 | EIF3B |
| 936 | PTBP1 |
| 937 | DNAJB2 |
| 938 | DDX58 |
| 939 | USP22 |
| 940 | CCNH |
| 941 | GART |
| 942 | LDHA |
| 943 | HADHB |
| 944 | VDAC1 |
| 945 | CORO1A |
| 946 | VDAC2 |
| 947 | CLIP1 |
| 948 | POMP |
| 949 | UGP2 |
| 950 | LDHB |
| 951 | CBFA2T2 |
| 952 | HIPK1 |
| 953 | MYO1B |
| 954 | COPB2 |
| 955 | MYO5A |
| 956 | CALCOCO1 |
| 957 | PCGF3 |
| 958 | EPHA4 |
| 959 | PPM1F |
| 960 | HSP90AB1 |
| 961 | CRYAB |
| 962 | STAT6 |
| 963 | EPB41L1 |
| 964 | BUB1 |
| 965 | MAPK10 |
| 966 | UBE2E3 |
| 967 | SORBS1 |
| 968 | RALBP1 |
| 969 | FGF12 |
| 970 | HSD17B4 |
| 971 | TREX1 |
| 972 | MGLL |
| 973 | CCAR1 |
| 974 | MPO |
| 975 | MPP1 |
| 976 | SRPR |
| 977 | HNRNPA0 |
| 978 | PRPF6 |
| 979 | AP2A1 |
| 980 | RASA1 |
| 981 | GPNMB |
| 982 | RPL37 |
| 983 | PRDX2 |
| 984 | PEX19 |
| 985 | CMC1 |
| 986 | ZBTB16 |
| 987 | PTN |
| 988 | GRLF1 |
| 989 | SUPT7L |
| 990 | DBH |
| 991 | EDN3 |
| 992 | KDM2B |
| 993 | CCT2 |
| 994 | DRG1 |
| 995 | ANXA4 |
| 996 | RPL26 |
| 997 | AK2 |
| 998 | ZNF423 |
| 999 | ZEB2 |
| 1000 | ZFPM2 |
| 1001 | PAXIP1 |
| 1002 | PCCA |
| 1003 | NKX3-1 |
| 1004 | TGFB1I1 |
| 1005 | PRDX3 |
| 1006 | HNMT |
| 1007 | OAZ2 |
| 1008 | TPX2 |
| 1009 | RC3H2 |
| 1010 | CHMP4B |
| 1011 | ITGB3 |
| 1012 | SRSF6 |
| 1013 | NIPBL |
| 1014 | PYGB |
| 1015 | MINK1 |
| 1016 | PEX14 |
| 1017 | TSC2 |
| 1018 | EIF2B4 |
| 1019 | ECT2 |
| 1020 | CCR7 |
| 1021 | DCHS1 |
| 1022 | ERH |
| 1023 | ARHGEF12 |
| 1024 | ARHGEF11 |
| 1025 | SNX3 |
| 1026 | SMURF1 |
| 1027 | PPP2CB |
| 1028 | NARS2 |
| 1029 | CETN2 |
| 1030 | RARB |
| 1031 | ADORA2A |
| 1032 | POGZ |
| 1033 | SGTA |
| 1034 | CCT7 |
| 1035 | BGN |
| 1036 | CIB1 |
| 1037 | HPN |
| 1038 | DLGAP4 |
| 1039 | MUL1 |
| 1040 | NTRK1 |
| 1041 | COL5A1 |
| 1042 | CCL2 |
| 1043 | ASH1L |
| 1044 | KRT1 |
| 1045 | RBP4 |
| 1046 | PTK7 |
| 1047 | ODF2 |
| 1048 | SPCS1 |
| 1049 | STIL |
| 1050 | SPARC |
| 1051 | PIK3CG |
| 1052 | KIDINS220 |
| 1053 | WWC1 |
| 1054 | MECP2 |
| 1055 | RBM12 |
| 1056 | RPL37A |
| 1057 | RBPMS |
| 1058 | FOLR1 |
| 1059 | ALDH3A2 |
| 1060 | PHYH |
| 1061 | SAFB |
| 1062 | KRT7 |
| 1063 | WNT7A |
| 1064 | AMPD2 |
| 1065 | WIPF1 |
| 1066 | NTRK2 |
| 1067 | HBM |
| 1068 | EIF2C1 |
| 1069 | CRHBP |
| 1070 | HYOU1 |
| 1071 | MDK |
| 1072 | ENAH |
| 1073 | STK4 |
| 1074 | ZMYND8 |
| 1075 | ZAK |
| 1076 | SSR1 |
| 1077 | EDA |
| 1078 | PKM2 |
| 1079 | HSPE1 |
| 1080 | HSPA4 |
| 1081 | DYNLL1 |
| 1082 | FSGS1 |
| 1083 | APC2 |
| 1084 | HSD17B12 |
| 1085 | HNRNPA1 |
| 1086 | EPRS |
| 1087 | GDI1 |
| 1088 | RNASET2 |
| 1089 | YY1 |
| 1090 | CHST3 |
| 1091 | NEBL |
| 1092 | WNT11 |
| 1093 | PSMF1 |
| 1094 | KDM1A |
| 1095 | NKX2-5 |
| 1096 | AGRN |
| 1097 | IVNS1ABP |
| 1098 | SPTLC1 |
| 1099 | PLEK |
| 1100 | SNRPB2 |
| 1101 | RNF114 |
| 1102 | TACC1 |
| 1103 | AP2M1 |
| 1104 | LRRC59 |
| 1105 | PPP1R16B |
| 1106 | HUWE1 |
| 1107 | DAZAP2 |
| 1108 | LRBA |
| 1109 | ZEB1 |
| 1110 | ESYT2 |
| 1111 | NAF1 |
| 1112 | PLP1 |
| 1113 | ZC3H12A |
| 1114 | TNK2 |
| 1115 | SPPL3 |
| 1116 | EHMT2 |
| 1117 | CUL2 |
| 1118 | TRIM24 |
| 1119 | EEF1A2 |
| 1120 | ANKRD28 |
| 1121 | WNT1 |
| 1122 | IFNGR1 |
| 1123 | OBSL1 |
| 1124 | DSG2 |
| 1125 | WRAP53 |
| 1126 | KIF11 |
| 1127 | TSNAX |
| 1128 | CCT6A |
| 1129 | TRMT112 |
| 1130 | RERE |
| 1131 | SYT7 |
| 1132 | GDNF |
| 1133 | SRSF2 |
| 1134 | PIK3CD |
| 1135 | PDS5A |
| 1136 | PRPF4 |
| 1137 | BTBD2 |
| 1138 | DLL1 |
| 1139 | BMPR1A |
| 1140 | CHSY1 |
| 1141 | SNURF |
| 1142 | USP3 |
| 1143 | DHCR24 |
| 1144 | HBEGF |
| 1145 | LGALS3 |
| 1146 | RPL38 |
| 1147 | UBA1 |
| 1148 | SNX17 |
| 1149 | MBD3 |
| 1150 | WNT3A |
| 1151 | CYLD |
| 1152 | HMGB3 |
| 1153 | POLR2A |
| 1154 | SERPINB9 |
| 1155 | ATP1A1 |
| 1156 | DPF2 |
| 1157 | BTG2 |
| 1158 | COL2A1 |
| 1159 | WNT4 |
| 1160 | KIF1A |
| 1161 | RNF213 |
| 1162 | GCH1 |
| 1163 | POLR2E |
| 1164 | M6PR |
| 1165 | RER1 |
| 1166 | BCAP31 |
| 1167 | IDDM20 |
| 1168 | SNAI2 |
| 1169 | COL5A2 |
| 1170 | CRY2 |
| 1171 | EDNRB |
| 1172 | TRIM25 |
| 1173 | CMDJ |
| 1174 | HIRA |
| 1175 | DROSHA |
| 1176 | ZNF335 |
| 1177 | YWHAG |
| 1178 | ATP6V0A1 |
| 1179 | HSBP1 |
| 1180 | PLCB4 |
| 1181 | ELOVL5 |
| 1182 | IARS |
| 1183 | AFG3L2 |
| 1184 | CHD7 |
| 1185 | ETM1 |
| 1186 | BCLAF1 |
| 1187 | AGT |
| 1188 | UBE3A |
| 1189 | MGEA5 |
| 1190 | SUMF1 |
| 1191 | PURA |
| 1192 | ZFP36 |
| 1193 | MBTPS2 |
| 1194 | EEA1 |
| 1195 | DRD4 |
| 1196 | ITPA |
| 1197 | CHP |
| 1198 | TGFB3 |
| 1199 | S100A9 |
| 1200 | DDB1 |
| 1201 | RAPGEF1 |
| 1202 | PPP1R12A |
| 1203 | NLRC5 |
| 1204 | TAF7 |
| 1205 | ANKH |
| 1206 | TRIM21 |
| 1207 | WHSC2 |
| 1208 | KIAA0562 |
| 1209 | HARS |
| 1210 | TAP2 |
| 1211 | AGXT2L2 |
| 1212 | PMEPA1 |
| 1213 | PRR1 |
| 1214 | RPL39 |
| 1215 | ATP5B |
| 1216 | RBM26 |
| 1217 | VCL |
| 1218 | SGPL1 |
| 1219 | DPAGT1 |
| 1220 | ZFP36L2 |
| 1221 | UBE2K |
| 1222 | TLK2 |
| 1223 | PPT1 |
| 1224 | SEPP1 |
| 1225 | ENY2 |
| 1226 | PINX1 |
| 1227 | SNAI1 |
| 1228 | CCND1 |
| 1229 | MST1 |
| 1230 | ATP2B1 |
| 1231 | NT5C2 |
| 1232 | HN1L |
| 1233 | AMD1 |
| 1234 | ITSN2 |
| 1235 | CSDE1 |
| 1236 | BDH2 |
| 1237 | DVL3 |
| 1238 | KIF1B |
| 1239 | ERLEC1 |
| 1240 | PLTP |
| 1241 | MCM5 |
| 1242 | CLTA |
| 1243 | ZMYND11 |
| 1244 | BLCAP |
| 1245 | MEFV |
| 1246 | EIF1 |
| 1247 | MYH9 |
| 1248 | ATP6V1H |
| 1249 | HMGN1 |
| 1250 | BCL2L1 |
| 1251 | GOLGB1 |
| 1252 | YME1L1 |
| 1253 | RPS21 |
| 1254 | TFCP2 |
| 1255 | MAPK8IP3 |
| 1256 | STK40 |
| 1257 | ATN1 |
| 1258 | VEZT |
| 1259 | ACP1 |
| 1260 | PIK3R4 |
| 1261 | ERGIC1 |
| 1262 | PAF1 |
| 1263 | INPP5D |
| 1264 | NBAS |
| 1265 | RANGAP1 |
| 1266 | AIMP2 |
| 1267 | RBBP4 |
| 1268 | KARS |
| 1269 | BTRC |
| 1270 | PAR1 |
| 1271 | MCRS1 |
| 1272 | ARRDC3 |
| 1273 | SUFU |
| 1274 | EDNRA |
| 1275 | RFC1 |
| 1276 | PJA2 |
| 1277 | PTMA |
| 1278 | LEMD2 |
| 1279 | SCNM1 |
| 1280 | ALMS1 |
| 1281 | SYNPO |
| 1282 | PARK2 |
| 1283 | GLO1 |
| 1284 | P4HB |
| 1285 | CSDA |
| 1286 | GNE |
| 1287 | MBOAT7 |
| 1288 | PPP1CC |
| 1289 | TSPYL1 |
| 1290 | LRRK2 |
| 1291 | UBASH3B |
| 1292 | CYC1 |
| 1293 | STRAP |
| 1294 | IL12A |
| 1295 | C14orf166 |
| 1296 | PRPF8 |
| 1297 | GNPAT |
| 1298 | PTGES3 |
| 1299 | TRPV4 |
| 1300 | BGLAP |
| 1301 | PRDM16 |
| 1302 | IGFBP7 |
| 1303 | DRD3 |
| 1304 | KAR |
| 1305 | QSOX1 |
| 1306 | ALDH1A3 |
| 1307 | UBE3B |
| 1308 | CHMP2A |
| 1309 | JAM3 |
| 1310 | C14orf145 |
| 1311 | AMACR |
| 1312 | FYN |
| 1313 | GNB2 |
| 1314 | AHR |
| 1315 | PRRC2B |
| 1316 | TAF10 |
| 1317 | BECN1 |
| 1318 | PEX2 |
| 1319 | SLC35A2 |
| 1320 | CUL7 |
| 1321 | MCM6 |
| 1322 | EGLN1 |
| 1323 | KPNA6 |
| 1324 | GAL |
| 1325 | MBTPS1 |
| 1326 | NEK2 |
| 1327 | NSDHL |
| 1328 | CCL5 |
| 1329 | TH1L |
| 1330 | RPGRIP1L |
| 1331 | TCEB2 |
| 1332 | CRP |
| 1333 | SETD5 |
| 1334 | GRSF1 |
| 1335 | HAMP |
| 1336 | TSHR |
| 1337 | PABPC4 |
| 1338 | ZNF451 |
| 1339 | APH1A |
| 1340 | RBFOX2 |
| 1341 | SRC |
| 1342 | HSD17B10 |
| 1343 | MCM3AP |
| 1344 | GDF5 |
| 1345 | ITGA6 |
| 1346 | VPS16 |
| 1347 | SND1 |
| 1348 | SPR |
| 1349 | ARPC5 |
| 1350 | RP33 |
| 1351 | NFIX |
| 1352 | DYNC1H1 |
| 1353 | IL1B |
| 1354 | CHI3L1 |
| 1355 | MGP |
| 1356 | PTPRK |
| 1357 | NEK6 |
| 1358 | EDN1 |
| 1359 | PDE7A |
| 1360 | EMP2 |
| 1361 | VPS28 |
| 1362 | AP2B1 |
| 1363 | ATP5L |
| 1364 | WNT10B |
| 1365 | SHANK3 |
| 1366 | VANGL2 |
| 1367 | DCLK1 |
| 1368 | TGFBR1 |
| 1369 | FAF1 |
| 1370 | CUL4A |
| 1371 | HMOX1 |
| 1372 | EIF3I |
| 1373 | KIF14 |
| 1374 | TRIM32 |
| 1375 | HLA-DQA1 |
| 1376 | SERPINA3 |
| 1377 | F2R |
| 1378 | COX7B |
| 1379 | DNAJA3 |
| 1380 | YWHAH |
| 1381 | OPHN1 |
| 1382 | UPF3B |
| 1383 | BTG1 |
| 1384 | DPYSL2 |
| 1385 | SMEK2 |
| 1386 | ANP32B |
| 1387 | PDGFA |
| 1388 | F5 |
| 1389 | COASY |
| 1390 | SORBS3 |
| 1391 | KANK2 |
| 1392 | OGG1 |
| 1393 | PREB |
| 1394 | KAT2B |
| 1395 | PCM1 |
| 1396 | KDM5B |
| 1397 | UBE2G2 |
| 1398 | PARK4 |
| 1399 | ATXN7 |
| 1400 | PRLR |
| 1401 | ADM |
| 1402 | PIAS4 |
| 1403 | NDUFA13 |
| 1404 | KRT18 |
| 1405 | IRF4 |
| 1406 | RBM4 |
| 1407 | NODAL |
| 1408 | NHP2L1 |
| 1409 | GLI3 |
| 1410 | NPNT |
| 1411 | MCM7 |
| 1412 | CLIC4 |
| 1413 | PLXNB2 |
| 1414 | ATP6V1B2 |
| 1415 | BCL10 |
| 1416 | XRCC3 |
| 1417 | LRPAP1 |
| 1418 | PKN1 |
| 1419 | PPP2R3C |
| 1420 | RFTN1 |
| 1421 | PDGFD |
| 1422 | HYAL2 |
| 1423 | SLC6A4 |
| 1424 | PXDN |
| 1425 | PTPRA |
| 1426 | RUNX1 |
| 1427 | LRPPRC |
| 1428 | NAMPT |
| 1429 | PLRG1 |
| 1430 | ERGIC3 |
| 1431 | PPP1R12B |
| 1432 | YWHAQ |
| 1433 | POLR2C |
| 1434 | PCGF2 |
| 1435 | NRIP1 |
| 1436 | PLXNB1 |
| 1437 | EIF2S2 |
| 1438 | DLL4 |
| 1439 | SKI |
| 1440 | KIAA0196 |
| 1441 | HINT1 |
| 1442 | IL1RN |
| 1443 | SEH1L |
| 1444 | LGALS3BP |
| 1445 | NOD2 |
| 1446 | VPS53 |
| 1447 | HAP1 |
| 1448 | SUMO2 |
| 1449 | KDM5A |
| 1450 | EIF3L |
| 1451 | RBM9 |
| 1452 | SCP2 |
| 1453 | MET |
| 1454 | DYNC1LI1 |
| 1455 | JAK1 |
| 1456 | MICAL2 |
| 1457 | ZFYVE26 |
| 1458 | DCAF6 |
| 1459 | AGL |
| 1460 | TNFSF4 |
| 1461 | CD27 |
| 1462 | TCERG1 |
| 1463 | ENTPD6 |
| 1464 | NUCKS1 |
| 1465 | RHEB |
| 1466 | TARS |
| 1467 | FAF2 |
| 1468 | GALC |
| 1469 | PMAIP1 |
| 1470 | ARPC2 |
| 1471 | MAP3K7 |
| 1472 | WSB1 |
| 1473 | ALOX12 |
| 1474 | GCDH |
| 1475 | VAV1 |
| 1476 | PLCG1 |
| 1477 | KAT5 |
| 1478 | PKN2 |
| 1479 | ZDHHC9 |
| 1480 | RAB8B |
| 1481 | GAS6 |
| 1482 | PMP22 |
| 1483 | SMARCAD1 |
| 1484 | TANK |
| 1485 | OPTN |
| 1486 | DCAF7 |
| 1487 | GATA4 |
| 1488 | PLA2G6 |
| 1489 | NALCN |
| 1490 | SLC39A1 |
| 1491 | SRI |
| 1492 | SOS2 |
| 1493 | EIF5 |
| 1494 | ITGB1BP1 |
| 1495 | HADHA |
| 1496 | ZC3H11A |
| 1497 | RANBP1 |
| 1498 | STOML2 |
| 1499 | PPP2R5E |
| 1500 | PITX2 |
| 1501 | RPL12 |
| 1502 | ATP7B |
| 1503 | NASP |
| 1504 | TRIP6 |
| 1505 | FSTL1 |
| 1506 | ISL1 |
| 1507 | FNTA |
| 1508 | SNRPN |
| 1509 | SLC8A1 |
| 1510 | TNFAIP3 |
| 1511 | TXNIP |
| 1512 | B3GALTL |
| 1513 | RAD51L1 |
| 1514 | RPL10A |
| 1515 | PTP4A1 |
| 1516 | SFRS4 |
| 1517 | SLC29A3 |
| 1518 | RYBP |
| 1519 | ETS1 |
| 1520 | ANO6 |
| 1521 | PRPF19 |
| 1522 | USP4 |
| 1523 | NUP133 |
| 1524 | C12orf5 |
| 1525 | S100A14 |
| 1526 | PPIE |
| 1527 | COL12A1 |
| 1528 | YARS2 |
| 1529 | XRN2 |
| 1530 | EYA1 |
| 1531 | IFI16 |
| 1532 | ADRBK1 |
| 1533 | SNAP25 |
| 1534 | PES1 |
| 1535 | HMGA2 |
| 1536 | CSNK2A2 |
| 1537 | MGMT |
| 1538 | CCDC47 |
| 1539 | ITSN1 |
| 1540 | PDLIM1 |
| 1541 | CUL4B |
| 1542 | ARFGAP2 |
| 1543 | PLCD1 |
| 1544 | CHCHD10 |
| 1545 | SLC22A5 |
| 1546 | WASH1 |
| 1547 | SLC25A13 |
| 1548 | HNRPDL |
| 1549 | BMIQ4 |
| 1550 | MGAT1 |
| 1551 | MC1R |
| 1552 | RNF220 |
| 1553 | ERP29 |
| 1554 | CPNE1 |
| 1555 | ARPP19 |
| 1556 | GTSE1 |
| 1557 | PRDX4 |
| 1558 | LGR4 |
| 1559 | GPLD1 |
| 1560 | GTF2F1 |
| 1561 | TFAP2B |
| 1562 | PPA1 |
| 1563 | SLC2A3 |
| 1564 | SENP5 |
| 1565 | SIK3 |
| 1566 | BMP1 |
| 1567 | PIK3R2 |
| 1568 | DLD |
| 1569 | CRBN |
| 1570 | LUM |
| 1571 | BAT2L1 |
| 1572 | LSM14A |
| 1573 | TNKS2 |
| 1574 | GOLGA4 |
| 1575 | MTSS1 |
| 1576 | UNC45A |
| 1577 | ATP7A |
| 1578 | CRTAP |
| 1579 | TGFB2 |
| 1580 | RPS29 |
| 1581 | FRAS1 |
| 1582 | WNT3 |
| 1583 | PAX3 |
| 1584 | POLE |
| 1585 | NAGA |
| 1586 | ZNF259 |
| 1587 | ELAVL1 |
| 1588 | MIB1 |
| 1589 | MAOA |
| 1590 | NUP85 |
| 1591 | RNF41 |
| 1592 | PRMT2 |
| 1593 | DTNB |
| 1594 | STN |
| 1595 | KIT |
| 1596 | UQCRC2 |
| 1597 | SUB1 |
| 1598 | PLXND1 |
| 1599 | SLC1A3 |
| 1600 | KDR |
| 1601 | CCL19 |
| 1602 | KIAA0528 |
| 1603 | SNX6 |
| 1604 | COL11A2 |
| 1605 | TGFA |
| 1606 | TAF15 |
| 1607 | TSR1 |
| 1608 | CLSTN1 |
| 1609 | MRFAP1 |
| 1610 | PHC2 |
| 1611 | ZBTB24 |
| 1612 | FTSJ1 |
| 1613 | KIF13B |
| 1614 | CYP27A1 |
| 1615 | ARPC3 |
| 1616 | SRRT |
| 1617 | AQP1 |
| 1618 | SMAD6 |
| 1619 | SPINK5 |
| 1620 | CLDN1 |
| 1621 | ALDH6A1 |
| 1622 | HLA-DRA |
| 1623 | SUCLG1 |
| 1624 | MOCS2 |
| 1625 | FAM123B |
| 1626 | SEC24C |
| 1627 | CSF2 |
| 1628 | SKIV2L2 |
| 1629 | ZFR |
| 1630 | GUCY1A3 |
| 1631 | CRMP1 |
| 1632 | TTR |
| 1633 | TAF2 |
| 1634 | THBS2 |
| 1635 | PCCB |
| 1636 | ITPKB |
| 1637 | WNT5A |
| 1638 | TYMS |
| 1639 | PRRC2A |
| 1640 | SUMO3 |
| 1641 | CPSF3L |
| 1642 | CCL21 |
| 1643 | CCT3 |
| 1644 | S100A10 |
| 1645 | PPM1B |
| 1646 | RCA1 |
| 1647 | PTGDS |
| 1648 | VIM |
| 1649 | SDHD |
| 1650 | S100A6 |
| 1651 | USP8 |
| 1652 | DSTN |
| 1653 | LGALS2 |
| 1654 | BAT2 |
| 1655 | CCR6 |
| 1656 | PRDX5 |
| 1657 | RPLP1 |
| 1658 | STRN3 |
| 1659 | PIP4K2B |
| 1660 | EIF2AK1 |
| 1661 | RBL2 |
| 1662 | STX16 |
| 1663 | FURIN |
| 1664 | CHD8 |
| 1665 | GATA1 |
| 1666 | QRICH1 |
| 1667 | ASPH |
| 1668 | MLX |
| 1669 | CLASP1 |
| 1670 | YWHAE |
| 1671 | LTBP2 |
| 1672 | ATXN10 |
| 1673 | DFN2 |
| 1674 | CLK2 |
| 1675 | PTPLAD1 |
| 1676 | KIAA1267 |
| 1677 | VAPA |
| 1678 | SMURF2 |
| 1679 | DYM |
| 1680 | DVL2 |
| 1681 | NCSTN |
| 1682 | SPRED1 |
| 1683 | AZIN1 |
| 1684 | GNS |
| 1685 | RSF1 |
| 1686 | CNA1 |
| 1687 | ZC3HAV1 |
| 1688 | LNX1 |
| 1689 | C14orf179 |
| 1690 | F2 |
| 1691 | PAK2 |
| 1692 | COL4A3BP |
| 1693 | HIF1AN |
| 1694 | SSRP1 |
| 1695 | MEB |
| 1696 | MYOCD |
| 1697 | BMP7 |
| 1698 | SPG20 |
| 1699 | MEN1 |
| 1700 | LIF |
| 1701 | ATP5F1 |
| 1702 | MLXIPL |
| 1703 | NSFL1C |
| 1704 | PHPT1 |
| 1705 | CACNA2D1 |
| 1706 | MID1 |
| 1707 | PTPN22 |
| 1708 | MPZL1 |
| 1709 | IVD |
| 1710 | TPD52L2 |
| 1711 | PPP2R5D |
| 1712 | RAB7A |
| 1713 | RBM25 |
| 1714 | ARL3 |
| 1715 | GPM6B |
| 1716 | KDELR1 |
| 1717 | RAB13 |
| 1718 | HTRA1 |
| 1719 | PRRC2C |
| 1720 | SPTBN1 |
| 1721 | DNMT3A |
| 1722 | MNAT1 |
| 1723 | SRSF1 |
| 1724 | TOR1A |
| 1725 | LTA4H |
| 1726 | SLC25A1 |
| 1727 | GAK |
| 1728 | BMPR2 |
| 1729 | CCR2 |
| 1730 | NOD1 |
| 1731 | RTN4 |
| 1732 | KCNJ11 |
| 1733 | CLASP2 |
| 1734 | DGCR2 |
| 1735 | CCR1 |
| 1736 | MED4 |
| 1737 | LRP4 |
| 1738 | RPRD1A |
| 1739 | SNAPIN |
| 1740 | KIFAP3 |
| 1741 | SLC2A1 |
| 1742 | TRPM7 |
| 1743 | WDR77 |
| 1744 | CEP290 |
| 1745 | CYR61 |
| 1746 | INPPL1 |
| 1747 | UFD1L |
| 1748 | DHX9 |
| 1749 | MAGED2 |
| 1750 | UNC119 |
| 1751 | SUGT1 |
| 1752 | ALDH18A1 |
| 1753 | TBX5 |
| 1754 | POLE3 |
| 1755 | TUBB4 |
| 1756 | ITGA5 |
| 1757 | MPHOSPH8 |
| 1758 | AHSA1 |
| 1759 | SYNCRIP |
| 1760 | LOX |
| 1761 | SUCLA2 |
| 1762 | CSF1R |
| 1763 | GLI2 |
| 1764 | PTPRF |
| 1765 | PRCC |
| 1766 | XPR1 |
| 1767 | FIG4 |
| 1768 | HERC1 |
| 1769 | FIS1 |
| 1770 | STXBP3 |
| 1771 | TGFBR2 |
| 1772 | VHL |
| 1773 | OAS1 |
| 1774 | MT3 |
| 1775 | IL16 |
| 1776 | U2AF1 |
| 1777 | FHL2 |
| 1778 | ALDH9A1 |
| 1779 | TRPS1 |
| 1780 | SLC7A7 |
| 1781 | TACSTD2 |
| 1782 | RPL32 |
| 1783 | SSR3 |
| 1784 | KIF1C |
| 1785 | CLEC16A |
| 1786 | RAB11FIP1 |
| 1787 | DKK1 |
| 1788 | COX6B1 |
| 1789 | AHCY |
| 1790 | BTK |
| 1791 | EVC2 |
| 1792 | BICD1 |
| 1793 | CCT4 |
| 1794 | GPR56 |
| 1795 | RPS7 |
| 1796 | MUSK |
| 1797 | GMDS |
| 1798 | SMAD3 |
| 1799 | SPOP |
| 1800 | UNC13A |
| 1801 | TXLNA |
| 1802 | BMP5 |
| 1803 | SPTLC2 |
| 1804 | FAM82A2 |
| 1805 | EPAS1 |
| 1806 | CRY1 |
| 1807 | EIF3M |
| 1808 | PARP4 |
| 1809 | MEOX1 |
| 1810 | TNPO1 |
| 1811 | VAMP3 |
| 1812 | SRA1 |
| 1813 | KDM6A |
| 1814 | RNF216 |
| 1815 | EXOC4 |
| 1816 | SMN2 |
| 1817 | DYX1C1 |
| 1818 | HAX1 |
| 1819 | RPS16 |
| 1820 | FBN1 |
| 1821 | PPP2R5C |
| 1822 | KPNA4 |
| 1823 | PPP2R4 |
| 1824 | PLEKHB2 |
| 1825 | PLK4 |
| 1826 | CTTN |
| 1827 | SVIL |
| 1828 | GPR26 |
| 1829 | TNFRSF11B |
| 1830 | HDAC8 |
| 1831 | PPIA |
| 1832 | DNMT3B |
| 1833 | SPEN |
| 1834 | GTF3C1 |
| 1835 | POLDIP3 |
| 1836 | ZRANB2 |
| 1837 | CP |
| 1838 | MORF4L2 |
| 1839 | PLK2 |
| 1840 | ERN1 |
| 1841 | ATP1A2 |
| 1842 | EIF3G |
| 1843 | ATP2B2 |
| 1844 | NLRP3 |
| 1845 | RBX1 |
| 1846 | EPO |
| 1847 | PRPF4B |
| 1848 | RARA |
| 1849 | CACYBP |
| 1850 | KIF3A |
| 1851 | GRK5 |
| 1852 | GNRH1 |
| 1853 | SYT17 |
| 1854 | PTGER4 |
| 1855 | TLR4 |
| 1856 | KAT2A |
| 1857 | SDF4 |
| 1858 | RPS12 |
| 1859 | PPIL2 |
| 1860 | NDUFS2 |
| 1861 | TROVE2 |
| 1862 | EXOSC8 |
| 1863 | PPIB |
| 1864 | AP3D1 |
| 1865 | GBP2 |
| 1866 | MYL12A |
| 1867 | PQBP1 |
| 1868 | F10 |
| 1869 | TANC2 |
| 1870 | OFC4 |
| 1871 | ATF3 |
| 1872 | FLNC |
| 1873 | BANF1 |
| 1874 | RUVBL1 |
| 1875 | GPS2 |
| 1876 | ETV6 |
| 1877 | EGLN3 |
| 1878 | IKZF1 |
| 1879 | RAB23 |
| 1880 | ESR2 |
| 1881 | DUSP3 |
| 1882 | ERO1LB |
| 1883 | KIAA0226 |
| 1884 | HNRNPA2B1 |
| 1885 | GP1BB |
| 1886 | GABARAP |
| 1887 | DPYSL3 |
| 1888 | ANKRD11 |
| 1889 | ATP6V1G1 |
| 1890 | LTBP4 |
| 1891 | SAT1 |
| 1892 | PAFAH1B1 |
| 1893 | NOL3 |
| 1894 | PWP1 |
| 1895 | NELF |
| 1896 | PPIF |
| 1897 | H19 |
| 1898 | ADORA1 |
| 1899 | PHLPP1 |
| 1900 | PTAFR |
| 1901 | ECM1 |
| 1902 | RIPK3 |
| 1903 | AP2A2 |
| 1904 | MDN1 |
| 1905 | PABPC1 |
| 1906 | PTRF |
| 1907 | ZMPSTE24 |
| 1908 | TPM1 |
| 1909 | TRIM28 |
| 1910 | OFD1 |
| 1911 | TRIM37 |
| 1912 | GOT1 |
| 1913 | IDH3B |
| 1914 | GJA1 |
| 1915 | DMXL2 |
| 1916 | IRF1 |
| 1917 | SARNP |
| 1918 | CDC73 |
| 1919 | KRIT1 |
| 1920 | ODZ4 |
| 1921 | NBR1 |
| 1922 | PNN |
| 1923 | C9orf3 |
| 1924 | SORT1 |
| 1925 | ARSB |
| 1926 | NOTCH2 |
| 1927 | RAB2A |
| 1928 | PAX2 |
| 1929 | RCN1 |
| 1930 | MMS19 |
| 1931 | SERPING1 |
| 1932 | ALAS1 |
| 1933 | ADRB2 |
| 1934 | AMCBX2 |
| 1935 | DHRS3 |
| 1936 | UBA3 |
| 1937 | PINK1 |
| 1938 | HM13 |
| 1939 | TSC1 |
| 1940 | EIF2S3 |
| 1941 | CRKL |
| 1942 | H2AFV |
| 1943 | TSN |
| 1944 | STK24 |
| 1945 | PCBP1 |
| 1946 | STC2 |
| 1947 | BBS2 |
| 1948 | PNPT1 |
| 1949 | MAN1B1 |
| 1950 | EFEMP2 |
| 1951 | MGST1 |
| 1952 | HERC2 |
| 1953 | GNAI2 |
| 1954 | IGFBP4 |
| 1955 | KIAA1279 |
| 1956 | DUSP1 |
| 1957 | LRS1 |
| 1958 | VEGFC |
| 1959 | OPRK1 |
| 1960 | DPP4 |
| 1961 | AFF4 |
| 1962 | KCNJ2 |
| 1963 | TPM2 |
| 1964 | RABGEF1 |
| 1965 | ASCL1 |
| 1966 | RPL8 |
| 1967 | PDLIM5 |
| 1968 | CMPK1 |
| 1969 | TRIM8 |
| 1970 | SELP |
| 1971 | MYH11 |
| 1972 | ARRB1 |
| 1973 | TWIST1 |
| 1974 | GBP1 |
| 1975 | CNC |
| 1976 | EIF2B2 |
| 1977 | CHID1 |
| 1978 | TBX3 |
| 1979 | STC1 |
| 1980 | ATG12 |
| 1981 | UNG |
| 1982 | PMPCA |
| 1983 | SH3BP5 |
| 1984 | ATG5 |
| 1985 | ANTXR2 |
| 1986 | MRPL3 |
| 1987 | KEAP1 |
| 1988 | SDHB |
| 1989 | SC5DL |
| 1990 | HLA-DPB1 |
| 1991 | CHST14 |
| 1992 | DRD1 |
| 1993 | GRIA1 |
| 1994 | CNOT1 |
| 1995 | UBE2A |
| 1996 | ITFG1 |
| 1997 | ANKRD10 |
| 1998 | ID4 |
| 1999 | BAP1 |
| 2000 | RAB5A |
| 2001 | PDXK |
| 2002 | RIPK1 |
| 2003 | MPDU1 |
| 2004 | SARS |
| 2005 | CHD4 |
| 2006 | THRA |
| 2007 | PTP4A2 |
| 2008 | MCC |
| 2009 | ALS2 |
| 2010 | ATP5E |
| 2011 | MALT1 |
| 2012 | VRK3 |
| 2013 | KLF10 |
| 2014 | RPS11 |
| 2015 | SYK |
| 2016 | PREX1 |
| 2017 | POSTN |
| 2018 | TADA3 |
| 2019 | ELF3 |
| 2020 | PNPLA6 |
| 2021 | SMARCC1 |
| 2022 | FOXL2 |
| 2023 | FUBP1 |
| 2024 | VASP |
| 2025 | TGFBR3 |
| 2026 | PTPN6 |
| 2027 | ADIPOR2 |
| 2028 | RTN3 |
| 2029 | FN1 |
| 2030 | VRK1 |
| 2031 | RPS28 |
| 2032 | BST2 |
| 2033 | MYO9B |
| 2034 | CDK9 |
| 2035 | RASSF1 |
| 2036 | CNTN4 |
| 2037 | PIK3C3 |
| 2038 | PSMA2 |
| 2039 | DSP |
| 2040 | EPB49 |
| 2041 | LRSAM1 |
| 2042 | BRE |
| 2043 | IL18 |
| 2044 | EIF4G3 |
| 2045 | IFT172 |
| 2046 | NEDD4 |
| 2047 | PORCN |
| 2048 | WIBG |
| 2049 | PLS3 |
| 2050 | CD2AP |
| 2051 | CPSF6 |
| 2052 | TUT1 |
| 2053 | SNX4 |
| 2054 | RPLP2 |
| 2055 | SRGN |
| 2056 | PRELID1 |
| 2057 | RPS13 |
| 2058 | SLX4 |
| 2059 | SPTBN2 |
| 2060 | TRA2B |
| 2061 | HDGF |
| 2062 | STT3B |
| 2063 | ZNHIT1 |
| 2064 | ATP5G3 |
| 2065 | CHD1L |
| 2066 | WDR34 |
| 2067 | RPS27 |
| 2068 | GNB5 |
| 2069 | NTSR1 |
| 2070 | HNRNPK |
| 2071 | NFAT5 |
| 2072 | RPS25 |
| 2073 | C1orf144 |
| 2074 | C1orf43 |
| 2075 | ATRX |
| 2076 | PFKP |
| 2077 | SMARCA5 |
| 2078 | MTA3 |
| 2079 | HNRNPM |
| 2080 | MAP2K1 |
| 2081 | MAPK11 |
| 2082 | SCAMP2 |
| 2083 | BID |
| 2084 | ALX4 |
| 2085 | CUL5 |
| 2086 | MKKS |
| 2087 | WIPI2 |
| 2088 | CENPJ |
| 2089 | SLC1A2 |
| 2090 | NAT15 |
| 2091 | CDK5 |
| 2092 | EEF1G |
| 2093 | VPS52 |
| 2094 | IKBKAP |
| 2095 | VAMP7 |
| 2096 | MFAP1 |
| 2097 | RPL17 |
| 2098 | ARHGEF7 |
| 2099 | HSPH1 |
| 2100 | INSIG1 |
| 2101 | TRPC6 |
| 2102 | ATP6AP1 |
| 2103 | NRBP1 |
| 2104 | CXCL14 |
| 2105 | P2RX4 |
| 2106 | UBAP2L |
| 2107 | MAPRE1 |
| 2108 | MORF4L1 |
| 2109 | AP3B1 |
| 2110 | POC1A |
| 2111 | LTA |
| 2112 | ADSS |
| 2113 | CLPTM1L |
| 2114 | PVR |
| 2115 | APPL1 |
| 2116 | TOB1 |
| 2117 | BRD7 |
| 2118 | THBS4 |
| 2119 | DBC1 |
| 2120 | HSP90AA2 |
| 2121 | SCD |
| 2122 | TNFRSF14 |
| 2123 | LGALS9 |
| 2124 | RCC2 |
| 2125 | PUM2 |
| 2126 | BAG3 |
| 2127 | AIS |
| 2128 | EEF2K |
| 2129 | RASGRP1 |
| 2130 | ID2 |
| 2131 | VCAM1 |
| 2132 | CD276 |
| 2133 | E2F4 |
| 2134 | RAC2 |
| 2135 | ZFAND3 |
| 2136 | USP10 |
| 2137 | STAT4 |
| 2138 | NDP |
| 2139 | GOLGA3 |
| 2140 | SPAST |
| 2141 | L1CAM |
| 2142 | TUBA1B |
| 2143 | RGS14 |
| 2144 | SERINC3 |
| 2145 | TH |
| 2146 | B4GALT7 |
| 2147 | ATP6V0D1 |
| 2148 | RPL5 |
| 2149 | NOV |
| 2150 | PKP2 |
| 2151 | TMEM135 |
| 2152 | SFRS6 |
| 2153 | DDX39 |
| 2154 | PCBD1 |
| 2155 | NEDD8 |
| 2156 | SH2B3 |
| 2157 | SNRNP200 |
| 2158 | ASCC2 |
| 2159 | AKR1B1 |
| 2160 | RAD23B |
| 2161 | RXRB |
| 2162 | PIK3C2A |
| 2163 | VPS35 |
| 2164 | RAB11A |
| 2165 | CMH3 |
| 2166 | UBE2D1 |
| 2167 | CCND2 |
| 2168 | SATB2 |
| 2169 | KLF4 |
| 2170 | SLC12A2 |
| 2171 | ROCK1 |
| 2172 | MED24 |
| 2173 | RXRA |
| 2174 | TNFSF10 |
| 2175 | AURKB |
| 2176 | VARS |
| 2177 | TAB1 |
| 2178 | UROS |
| 2179 | TMED10 |
| 2180 | GCK |
| 2181 | TALDO1 |
| 2182 | PYCARD |
| 2183 | RRM2 |
| 2184 | SUPT16H |
| 2185 | LRP1 |
| 2186 | MEGF8 |
| 2187 | SPAG9 |
| 2188 | TENC1 |
| 2189 | KLF6 |
| 2190 | DDX23 |
| 2191 | NR5A1 |
| 2192 | SLC7A5 |
| 2193 | ATP5C1 |
| 2194 | NEFL |
| 2195 | SEC23B |
| 2196 | MTA1 |
| 2197 | HNF1B |
| 2198 | RAB5B |
| 2199 | FXR2 |
| 2200 | EIF2AK4 |
| 2201 | CSRP1 |
| 2202 | CFH |
| 2203 | DVL1 |
| 2204 | CARM1 |
| 2205 | HLA-DPA1 |
| 2206 | NLK |
| 2207 | FOXP3 |
| 2208 | FBL |
| 2209 | AGTR2 |
| 2210 | SMOC1 |
| 2211 | G6PC3 |
| 2212 | AIF1 |
| 2213 | ASAH1 |
| 2214 | SOCS5 |
| 2215 | DOCK1 |
| 2216 | HDLBP |
| 2217 | B3GALT6 |
| 2218 | MSH2 |
| 2219 | SBDS |
| 2220 | BRI3 |
| 2221 | AXL |
| 2222 | PHLDB2 |
| 2223 | NCOA2 |
| 2224 | MLL4 |
| 2225 | CDKN2C |
| 2226 | PSMA3 |
| 2227 | EIF2AK3 |
| 2228 | LZTS1 |
| 2229 | HLA-DRB5 |
| 2230 | EIF3D |
| 2231 | PGM1 |
| 2232 | SLC37A4 |
| 2233 | ATF4C |
| 2234 | HCFC1 |
| 2235 | SNX9 |
| 2236 | BCAR3 |
| 2237 | THOC2 |
| 2238 | TRIB1 |
| 2239 | CYBB |
| 2240 | BAZ1A |
| 2241 | SELE |
| 2242 | KIAA0146 |
| 2243 | DDAH1 |
| 2244 | BNIP3 |
| 2245 | IWS1 |
| 2246 | SETX |
| 2247 | TRPM4 |
| 2248 | MAPK6 |
| 2249 | NAE1 |
| 2250 | HEYL |
| 2251 | BMP2 |
| 2252 | SIX3 |
| 2253 | COL1A2 |
| 2254 | RPTOR |
| 2255 | GJB6 |
| 2256 | LEMD3 |
| 2257 | FBXW7 |
| 2258 | TRIM44 |
| 2259 | DDX24 |
| 2260 | RPS15A |
| 2261 | ANP32A |
| 2262 | CXCL12 |
| 2263 | STS |
| 2264 | CTDSP2 |
| 2265 | H2AFY |
| 2266 | ANTXR1 |
| 2267 | HNRNPA3 |
| 2268 | GNB2L1 |
| 2269 | IFIT1 |
| 2270 | SH3GLB1 |
| 2271 | WDR1 |
| 2272 | CMTM6 |
| 2273 | JAG1 |
| 2274 | SP100 |
| 2275 | SLC6A8 |
| 2276 | UBL5 |
| 2277 | KRT17 |
| 2278 | GJB2 |
| 2279 | DNAJA1 |
| 2280 | CHD3 |
| 2281 | LMO2 |
| 2282 | MRE11A |
| 2283 | TRIB2 |
| 2284 | SOX17 |
| 2285 | RPS26 |
| 2286 | ZNF24 |
| 2287 | SFRP2 |
| 2288 | ALAS2 |
| 2289 | XRCC4 |
| 2290 | PLOD3 |
| 2291 | SEC24D |
| 2292 | KIF3B |
| 2293 | JPD |
| 2294 | SMARCA4 |
| 2295 | ATIC |
| 2296 | GNAI3 |
| 2297 | RAB14 |
| 2298 | ZYG11B |
| 2299 | WDFY3 |
| 2300 | PSME4 |
| 2301 | KRT5 |
| 2302 | BCKDHA |
| 2303 | PSMD1 |
| 2304 | AHCYL1 |
| 2305 | ANXA2P3 |
| 2306 | CHUK |
| 2307 | PSMB6 |
| 2308 | PLA2G1B |
| 2309 | MYH10 |
| 2310 | EPS15 |
| 2311 | GNPTAB |
| 2312 | IDS |
| 2313 | CD40LG |
| 2314 | PRDM1 |
| 2315 | PEBP1 |
| 2316 | BAZ2A |
| 2317 | SHOC2 |
| 2318 | SNX14 |
| 2319 | RPL18 |
| 2320 | PYGL |
| 2321 | GATA2 |
| 2322 | MDH1 |
| 2323 | SOX4 |
| 2324 | SF3B1 |
| 2325 | GNB1 |
| 2326 | DES |
| 2327 | KIAA0368 |
| 2328 | RPL4 |
| 2329 | DYNC1LI2 |
| 2330 | GOLGA1 |
| 2331 | ARHGDIA |
| 2332 | SERBP1 |
| 2333 | NSUN2 |
| 2334 | MYLIP |
| 2335 | BIN1 |
| 2336 | QDPR |
| 2337 | WASL |
| 2338 | AGA |
| 2339 | KIAA0430 |
| 2340 | C12orf57 |
| 2341 | COL6A2 |
| 2342 | LGALS1 |
| 2343 | SULF1 |
| 2344 | PPP3CA |
| 2345 | CTNS |
| 2346 | CUL3 |
| 2347 | VTN |
| 2348 | RCN2 |
| 2349 | SRP72 |
| 2350 | MFGE8 |
| 2351 | GPC6 |
| 2352 | SFRS18 |
| 2353 | DPM1 |
| 2354 | ADIPOQ |
| 2355 | VPS33B |
| 2356 | RAG1 |
| 2357 | WBP4 |
| 2358 | NAV2 |
| 2359 | YAP1 |
| 2360 | FNBP1 |
| 2361 | ATG16L1 |
| 2362 | KIFC3 |
| 2363 | PRNP |
| 2364 | BAG2 |
| 2365 | LITAF |
| 2366 | RAGE |
| 2367 | POLR1A |
| 2368 | C5orf13 |
| 2369 | ARG1 |
| 2370 | ARFGEF2 |
| 2371 | BYSL |
| 2372 | IL23R |
| 2373 | CDC123 |
| 2374 | TNIK |
| 2375 | IL1A |
| 2376 | TLE1 |
| 2377 | SRP9 |
| 2378 | MAP2K6 |
| 2379 | PPAP2B |
| 2380 | PIGT |
| 2381 | GLS |
| 2382 | GMPPA |
| 2383 | COL3A1 |
| 2384 | ARF3 |
| 2385 | BCL3 |
| 2386 | TMED2 |
| 2387 | TSPO |
| 2388 | RAB6C |
| 2389 | UBE3C |
| 2390 | MX1 |
| 2391 | SPI1 |
| 2392 | IRAK1 |
| 2393 | BARD1 |
| 2394 | BIRC3 |
| 2395 | EMG1 |
| 2396 | YTHDF2 |
| 2397 | SERINC1 |
| 2398 | HDAC5 |
| 2399 | MYD88 |
| 2400 | RNF31 |
| 2401 | CPX |
| 2402 | TUBB2C |
| 2403 | APOC1 |
| 2404 | EIF2C2 |
| 2405 | WHSC1 |
| 2406 | SCPEP1 |
| 2407 | PPP1R9A |
| 2408 | SSR4 |
| 2409 | PRRX1 |
| 2410 | IGHD |
| 2411 | SNRNP70 |
| 2412 | CFTR |
| 2413 | TRPC7 |
| 2414 | C10orf2 |
| 2415 | CDC25B |
| 2416 | SDS |
| 2417 | VAV2 |
| 2418 | HLA-A |
| 2419 | SAMM50 |
| 2420 | AKAP6 |
| 2421 | RPS8 |
| 2422 | WAPAL |
| 2423 | RPL31 |
| 2424 | FUZ |
| 2425 | IHG1 |
| 2426 | EMP1 |
| 2427 | ERRFI1 |
| 2428 | SLC11A1 |
| 2429 | OMG |
| 2430 | TNRC6B |
| 2431 | SLC38A2 |
| 2432 | SPHK1 |
| 2433 | APOA1 |
| 2434 | CITED2 |
| 2435 | GPC3 |
| 2436 | CTNNBL1 |
| 2437 | PRKAA2 |
| 2438 | CHEK1 |
| 2439 | AKR1A1 |
| 2440 | DDX3X |
| 2441 | CCNG1 |
| 2442 | GPHN |
| 2443 | MAP3K1 |
| 2444 | HPRT1 |
| 2445 | OPRM1 |
| 2446 | PRPF3 |
| 2447 | NOS2 |
| 2448 | POLD2 |
| 2449 | FBP1 |
| 2450 | SF3B3 |
| 2451 | RBM5 |
| 2452 | LRRFIP1 |
| 2453 | RPAIN |
| 2454 | MTR |
| 2455 | EGLN2 |
| 2456 | MTDH |
| 2457 | HTR2B |
| 2458 | NDUFB8 |
| 2459 | RPL18A |
| 2460 | FMR1 |
| 2461 | IGF2BP2 |
| 2462 | MAGOH |
| 2463 | ATP1B1 |
| 2464 | MAP3K14 |
| 2465 | RIPK4 |
| 2466 | TCAP |
| 2467 | MSN |
| 2468 | DIS3L2 |
| 2469 | RAB1B |
| 2470 | MTRR |
| 2471 | TBL1X |
| 2472 | ADRM1 |
| 2473 | TBK1 |
| 2474 | EGR2 |
| 2475 | RYR1 |
| 2476 | CNTF |
| 2477 | MEF2D |
| 2478 | NUDT3 |
| 2479 | UQCRQ |
| 2480 | PPP2R2B |
| 2481 | TRPM2 |
| 2482 | ZFP106 |
| 2483 | SEMA3E |
| 2484 | CD2BP2 |
| 2485 | IPO4 |
| 2486 | CEP55 |
| 2487 | RBM10 |
| 2488 | CYP27B1 |
| 2489 | CTBP1 |
| 2490 | PTTG1IP |
| 2491 | SLC25A12 |
| 2492 | DLAT |
| 2493 | NAA10 |
| 2494 | ARF6 |
| 2495 | XPO5 |
| 2496 | PAK3 |
| 2497 | MTMR4 |
| 2498 | WDR19 |
| 2499 | SPG7 |
| 2500 | MKRN1 |
| 2501 | RBMX |
| 2502 | CBR1 |
| 2503 | GRIN1 |
| 2504 | SH3KBP1 |
| 2505 | COX6A1 |
| 2506 | FOXC1 |
| 2507 | NDRG1 |
| 2508 | TIMP1 |
| 2509 | SCO1 |
| 2510 | NOP58 |
| 2511 | PDHB |
| 2512 | TTC17 |
| 2513 | TUBG1 |
| 2514 | ALDOA |
| 2515 | DOCK8 |
| 2516 | DTNBP1 |
| 2517 | KIF5A |
| 2518 | PLDN |
| 2519 | CNBP |
| 2520 | CTSL1 |
| 2521 | RCOR1 |
| 2522 | STARD7 |
| 2523 | ZFAND5 |
| 2524 | RPRD2 |
| 2525 | CHGA |
| 2526 | STK25 |
| 2527 | ATP1A3 |
| 2528 | COPS2 |
| 2529 | SNW1 |
| 2530 | NPC1 |
| 2531 | SHFM1 |
| 2532 | HLA-DRB4 |
| 2533 | ARFIP2 |
| 2534 | CD109 |
| 2535 | CARS |
| 2536 | INPP5E |
| 2537 | SOX2 |
| 2538 | CD63 |
| 2539 | RPS17 |
| 2540 | MKNK2 |
| 2541 | BIRC5 |
| 2542 | PHGDH |
| 2543 | APLP2 |
| 2544 | PSMC2 |
| 2545 | ATAD1 |
| 2546 | TMEM9 |
| 2547 | MED12 |
| 2548 | SEMA5A |
| 2549 | EIF2B1 |
| 2550 | BLZF1 |
| 2551 | TKT |
| 2552 | SMAD1 |
| 2553 | TNFAIP1 |
| 2554 | SLC25A5 |
| 2555 | CPLX1 |
| 2556 | RAB6A |
| 2557 | WDR5 |
| 2558 | PIBF1 |
| 2559 | C2orf47 |
| 2560 | RELN |
| 2561 | EIF3F |
| 2562 | MYH7 |
| 2563 | VPS4A |
| 2564 | CD164 |
| 2565 | HMGN2 |
| 2566 | LMO4 |
| 2567 | KIAA1310 |
| 2568 | WTS |
| 2569 | CYCS |
| 2570 | SNIP1 |
| 2571 | EIF4H |
| 2572 | ATXN2 |
| 2573 | ECH1 |
| 2574 | SMAD2 |
| 2575 | STAG3L3 |
| 2576 | YWHAB |
| 2577 | S1PR1 |
| 2578 | SLC38A1 |
| 2579 | HTR2A |
| 2580 | MYCN |
| 2581 | RBM3 |
| 2582 | UROD |
| 2583 | SLC9A1 |
| 2584 | BASP1 |
| 2585 | APOB |
| 2586 | PRKAB1 |
| 2587 | COMMD1 |
| 2588 | NSD1 |
| 2589 | DKC1 |
| 2590 | ATP2A2 |
| 2591 | SON |
| 2592 | ESCO2 |
| 2593 | PER1 |
| 2594 | PTPRO |
| 2595 | DRP2 |
| 2596 | SNCA |
| 2597 | RAB10 |
| 2598 | TRPC4AP |
| 2599 | NDUFA9 |
| 2600 | FOXP1 |
| 2601 | YPEL5 |
| 2602 | CDK5RAP2 |
| 2603 | GNA12 |
| 2604 | PLN |
| 2605 | CADM1 |
| 2606 | RALB |
| 2607 | SEC14L1 |
| 2608 | MRPL44 |
| 2609 | POLR2G |
| 2610 | TRIM27 |
| 2611 | ROBO2 |
| 2612 | GIT2 |
| 2613 | MOV10 |
| 2614 | RPL34 |
| 2615 | WDR35 |
| 2616 | RPL13A |
| 2617 | ITGA3 |
| 2618 | LRP10 |
| 2619 | HBXIP |
| 2620 | LAMP2 |
| 2621 | IMPAD1 |
| 2622 | PLOD1 |
| 2623 | ATP6AP2 |
| 2624 | PTPN23 |
| 2625 | KDSR |
| 2626 | RARG |
| 2627 | LAPTM4A |
| 2628 | SLC25A11 |
| 2629 | CD28 |
| 2630 | GSTK1 |
| 2631 | RAPGEF3 |
| 2632 | PFKL |
| 2633 | SFPQ |
| 2634 | TBX1 |
| 2635 | NIN |
| 2636 | PDIA4 |
| 2637 | SSBP1 |
| 2638 | CLK1 |
| 2639 | LIFR |
| 2640 | FGF8 |
| 2641 | TK2 |
| 2642 | LARGE |
| 2643 | TNFRSF21 |
| 2644 | TMOD1 |
| 2645 | NDUFA1 |
| 2646 | TOMM34 |
| 2647 | FUS |
| 2648 | PXK |
| 2649 | RAB18 |
| 2650 | CTSH |
| 2651 | NCAM1 |
| 2652 | FES |
| 2653 | COPS8 |
| 2654 | CDK6 |
| 2655 | SMARCB1 |
| 2656 | VPS13B |
| 2657 | TOM1L1 |
| 2658 | TPR |
| 2659 | IGF2R |
| 2660 | UCN |
| 2661 | FAM20C |
| 2662 | DDC |
| 2663 | C5orf32 |
| 2664 | TAPBP |
| 2665 | NCOA1 |
| 2666 | PSMB1 |
| 2667 | PFKFB3 |
| 2668 | E2F3 |
| 2669 | RAB3GAP2 |
| 2670 | NOP56 |
| 2671 | FGF10 |
| 2672 | CALR |
| 2673 | SRSF4 |
| 2674 | VLDLR |
| 2675 | STK38 |
| 2676 | TUBB1 |
| 2677 | DNM1 |
| 2678 | LONP2 |
| 2679 | PLG |
| 2680 | MSX2 |
| 2681 | BCL2L11 |
| 2682 | GTF3C2 |
| 2683 | COL11A1 |
| 2684 | PCSK5 |
| 2685 | EIF5B |
| 2686 | ASNA1 |
| 2687 | SOX9 |
| 2688 | TMED9 |
| 2689 | TBCE |
| 2690 | CTPS |
| 2691 | POU5F1 |
| 2692 | TNFRSF1B |
| 2693 | GPX3 |
| 2694 | CDK2 |
| 2695 | ALB |
| 2696 | VAPB |
| 2697 | ICAM1 |
| 2698 | PRKAB2 |
| 2699 | PARK7 |
| 2700 | XYLT1 |
| 2701 | STAG2 |
| 2702 | GNG12 |
| 2703 | NOLC1 |
| 2704 | FLI1 |
| 2705 | GNAQ |
| 2706 | EWSR1 |
| 2707 | DYNC2LI1 |
| 2708 | SLC25A3 |
| 2709 | SF3A3 |
| 2710 | TP53BP2 |
| 2711 | BBS4 |
| 2712 | CTNNBIP1 |
| 2713 | VWF |
| 2714 | LMAN2 |
| 2715 | TAP1 |
| 2716 | APAF1 |
| 2717 | SPTAN1 |
| 2718 | ELAC2 |
| 2719 | MAPK7 |
| 2720 | MOGS |
| 2721 | PPP1R7 |
| 2722 | C6orf125 |
| 2723 | TK1 |
| 2724 | NDST1 |
| 2725 | CSE1L |
| 2726 | TMEM49 |
| 2727 | GPS1 |
| 2728 | DYSF |
| 2729 | RAP1BL |
| 2730 | DISC1 |
| 2731 | PNKD |
| 2732 | SOX11 |
| 2733 | NAPA |
| 2734 | FNDC3B |
| 2735 | RREB1 |
| 2736 | AP1M1 |
| 2737 | TEK |
| 2738 | RUVBL2 |
| 2739 | TXNDC5 |
| 2740 | MMP9 |
| 2741 | CAPZB |
| 2742 | TOMM20 |
| 2743 | HRX |
| 2744 | CD3E |
| 2745 | GBE1 |
| 2746 | YKT6 |
| 2747 | FOSL1 |
| 2748 | GUSB |
| 2749 | PLEKHA5 |
| 2750 | GYS1 |
| 2751 | NPHP1 |
| 2752 | SF3A1 |
| 2753 | CCM2 |
| 2754 | IFNG |
| 2755 | CSPP1 |
| 2756 | FLOT2 |
| 2757 | FOXC2 |
| 2758 | SLC9A3R1 |
| 2759 | MUT |
| 2760 | JBTS1 |
| 2761 | PPAT |
| 2762 | CNTN2 |
| 2763 | CCR5 |
| 2764 | MAN2A2 |
| 2765 | PCBP2 |
| 2766 | C19orf2 |
| 2767 | AUH |
| 2768 | ENO2 |
| 2769 | PEF1 |
| 2770 | CHRM3 |
| 2771 | PMPCB |
| 2772 | TUBGCP4 |
| 2773 | MEF2C |
| 2774 | OAT |
| 2775 | MAP3K3 |
| 2776 | MPRIP |
| 2777 | FLAD1 |
| 2778 | RPS9 |
| 2779 | BPTF |
| 2780 | C1QBP |
| 2781 | WDR45L |
| 2782 | BAT2L2 |
| 2783 | BMP6 |
| 2784 | NPPA |
| 2785 | FBLN5 |
| 2786 | PIAS1 |
| 2787 | SFRS3 |
| 2788 | EPHA2 |
| 2789 | AKT3 |
| 2790 | ALDH7A1 |
| 2791 | NLGN1 |
| 2792 | NONO |
| 2793 | SQRDL |
| 2794 | PTPN3 |
| 2795 | RUNX2 |
| 2796 | DDX41 |
| 2797 | COL1A1 |
| 2798 | DNAJA2 |
| 2799 | UQCRC1 |
| 2800 | PPP2R1B |
| 2801 | SAP18 |
| 2802 | MYL12B |
| 2803 | ITGA4 |
| 2804 | ILK |
| 2805 | GTF3C3 |
| 2806 | CUBN |
| 2807 | GPER |
| 2808 | MAP1LC3A |
| 2809 | MLLT6 |
| 2810 | MAP1B |
| 2811 | CDH13 |
| 2812 | CCDC22 |
| 2813 | TTK |
| 2814 | CIAO1 |
| 2815 | SAR1B |
| 2816 | CDK16 |
| 2817 | NFKBIB |
| 2818 | TTC37 |
| 2819 | IL2RA |
| 2820 | FBN2 |
| 2821 | HMGCL |
| 2822 | TAC1 |
| 2823 | SIX1 |
| 2824 | SETBP1 |
| 2825 | CALCOCO2 |
| 2826 | EFNA1 |
| 2827 | PSMB7 |
| 2828 | AKAP9 |
| 2829 | RNF4 |
| 2830 | VTA1 |
| 2831 | STOM |
| 2832 | CFLAR |
| 2833 | RRM2B |
| 2834 | MBL2 |
| 2835 | MED13 |
| 2836 | LAMA3 |
| 2837 | RIPK2 |
| 2838 | HIP1 |
| 2839 | SH3PXD2B |
| 2840 | STX3 |
| 2841 | CBARA1 |
| 2842 | FNDC3A |
| 2843 | EFNB1 |
| 2844 | TTC1 |
| 2845 | KIAA1949 |
| 2846 | TRAF4 |
| 2847 | DCN |
| 2848 | ODC1 |
| 2849 | CST3 |
| 2850 | ECHS1 |
| 2851 | TMEM43 |
| 2852 | SMARCD1 |
| 2853 | C5 |
| 2854 | CLPTM1 |
| 2855 | PSMB5 |
| 2856 | RNF10 |
| 2857 | CTSS |
| 2858 | DEAF1 |
| 2859 | YIPF3 |
| 2860 | FTH1 |
| 2861 | USP14 |
| 2862 | SERP1 |
| 2863 | F11R |
| 2864 | SHB |
| 2865 | CBX1 |
| 2866 | MKS1 |
| 2867 | TRAF3IP1 |
| 2868 | ID1 |
| 2869 | LIG3 |
| 2870 | SCO2 |
| 2871 | VPS11 |
| 2872 | HNRNPH2 |
| 2873 | UVRAG |
| 2874 | PCNT |
| 2875 | PAG1 |
| 2876 | CTSB |
| 2877 | NCK2 |
| 2878 | TPT1 |
| 2879 | TAZ |
| 2880 | EIF2B5 |
| 2881 | RTEL1 |
| 2882 | NRP2 |
| 2883 | KCNH1 |
| 2884 | HFE |
| 2885 | PSMC4 |
| 2886 | RPL35A |
| 2887 | WDR73 |
| 2888 | EIF4G1 |
| 2889 | GLUD1 |
| 2890 | C1QTNF3 |
| 2891 | ANXA6 |
| 2892 | SF3B4 |
| 2893 | EXT1 |
| 2894 | CDC16 |
| 2895 | SLC33A1 |
| 2896 | DNM2 |
| 2897 | MFN2 |
| 2898 | INHBA |
| 2899 | TRIO |
| 2900 | TCTN3 |
| 2901 | PSME2 |
| 2902 | RAB35 |
| 2903 | VAV3 |
| 2904 | DYNLT1 |
| 2905 | TMEM165 |
| 2906 | CORO1B |
| 2907 | GPD1 |
| 2908 | C2orf28 |
| 2909 | C20orf72 |
| 2910 | PAPSS1 |
| 2911 | CD4 |
| 2912 | SLC6A3 |
| 2913 | AUP1 |
| 2914 | RAB31 |
| 2915 | GNAI1 |
| 2916 | CD14 |
| 2917 | PXN |
| 2918 | RFC3 |
| 2919 | CHCHD2 |
| 2920 | DNAJB6 |
| 2921 | ENPP2 |
| 2922 | SF3B2 |
| 2923 | RPS14 |
| 2924 | PUF60 |
| 2925 | S100A11 |
| 2926 | ROD1 |
| 2927 | GOPC |
| 2928 | AKAP12 |
| 2929 | MAP2K7 |
| 2930 | NRAS |
| 2931 | TIRAP |
| 2932 | ITGA2 |
| 2933 | ARL6IP5 |
| 2934 | HADH |
| 2935 | TRAF1 |
| 2936 | PHB2 |
| 2937 | NOTCH1 |
| 2938 | KDM2A |
| 2939 | C1orf63 |
| 2940 | RBM14 |
| 2941 | COPZ1 |
| 2942 | MRPS16 |
| 2943 | MUTED |
| 2944 | RANBP2 |
| 2945 | METTL3 |
| 2946 | OGDH |
| 2947 | MTM1 |
| 2948 | PEX7 |
| 2949 | ATF5 |
| 2950 | MBD5 |
| 2951 | INO80 |
| 2952 | HIP1R |
| 2953 | SOX10 |
| 2954 | ALDH5A1 |
| 2955 | BZW1 |
| 2956 | SRSF3 |
| 2957 | RPN1 |
| 2958 | TTC8 |
| 2959 | NGFRAP1 |
| 2960 | GSPT1 |
| 2961 | IFIH1 |
| 2962 | C10orf58 |
| 2963 | CD96 |
| 2964 | ILK-2 |
| 2965 | PER2 |
| 2966 | PSAP |
| 2967 | WDYHV1 |
| 2968 | HLCS |
| 2969 | UBQLN1 |
| 2970 | DKK3 |
| 2971 | LETM1 |
| 2972 | LUZP1 |
| 2973 | YBX1 |
| 2974 | LTF |
| 2975 | PEPD |
| 2976 | CECR1 |
| 2977 | GSTM3 |
| 2978 | C2 |
| 2979 | CENPE |
| 2980 | FARSB |
| 2981 | HRD |
| 2982 | PRKAA1 |
| 2983 | TMSB10 |
| 2984 | FGF14 |
| 2985 | PSMD8 |
| 2986 | AIRE |
| 2987 | COPE |
| 2988 | TGM2 |
| 2989 | UBE2Z |
| 2990 | COQ9 |
| 2991 | SMS |
| 2992 | NF2 |
| 2993 | IRAK3 |
| 2994 | CDH12 |
| 2995 | GSTO1 |
| 2996 | KHDRBS1 |
| 2997 | CTLA4 |
| 2998 | CCL3 |
| 2999 | NDC80 |
| 3000 | FGF1 |
| 3001 | CBL |
| 3002 | UHRF2 |
| 3003 | SCAP |
| 3004 | TRA2A |
| 3005 | LAMP1 |
| 3006 | PITRM1 |
| 3007 | SYPL1 |
| 3008 | TMOD3 |
| 3009 | POMC |
| 3010 | GLTSCR2 |
| 3011 | UBQLN4 |
| 3012 | CCNC |
| 3013 | CDKN1B |
| 3014 | RPL35 |
| 3015 | RPL19 |
| 3016 | ATF6 |
| 3017 | ITPR1 |
| 3018 | IL6R |
| 3019 | ZNF587 |
| 3020 | COPG |
| 3021 | BAG1 |
| 3022 | C17orf68 |
| 3023 | RNASEN |
| 3024 | ARL8B |
| 3025 | B4GALT1 |
| 3026 | MKRN3 |
| 3027 | GORASP2 |
| 3028 | RPL36 |
| 3029 | SUV39H1 |
| 3030 | RHOB |
| 3031 | SCARB1 |
| 3032 | KLHDC3 |
| 3033 | IMMT |
| 3034 | C9orf31 |
| 3035 | OXTR |
| 3036 | EIF1B |
| 3037 | MAN2B1 |
| 3038 | IL8 |
| 3039 | CFL2 |
| 3040 | IL33 |
| 3041 | RNF11 |
| 3042 | ARHGDIB |
| 3043 | KFSD |
| 3044 | CDC27 |
| 3045 | DGKZ |
| 3046 | NDUFV2 |
| 3047 | CLN8 |
| 3048 | DCTN2 |
| 3049 | C13orf15 |
| 3050 | KDM4A |
| 3051 | FAM59A |
| 3052 | LMAN1 |
| 3053 | PSMB8 |
| 3054 | UBXN1 |
| 3055 | MEF2A |
| 3056 | GLRX3 |
| 3057 | SKP1 |
| 3058 | TLR9 |
| 3059 | ALPL |
| 3060 | IPO7 |
| 3061 | HCK |
| 3062 | CD40 |
| 3063 | SSU72 |
| 3064 | CDC37 |
| 3065 | TSG101 |
| 3066 | MSX1 |
| 3067 | FANCD2 |
| 3068 | LAPTM4B |
| 3069 | H1F0 |
| 3070 | CEP70 |
| 3071 | CUL1 |
| 3072 | CPSF1 |
| 3073 | PIGA |
| 3074 | UHMK1 |
| 3075 | EIF4ENIF1 |
| 3076 | MTHFD2 |
| 3077 | TRAF2 |
| 3078 | FOXF1 |
| 3079 | UGP1 |
| 3080 | KPNB1 |
| 3081 | PPP1R8 |
| 3082 | GSN |
| 3083 | USP33 |
| 3084 | CD38 |
| 3085 | PPP3CB |
| 3086 | IL4 |
| 3087 | HDAC4 |
| 3088 | DNASE1L1 |
| 3089 | HRG |
| 3090 | PSMA7 |
| 3091 | MLL |
| 3092 | CRK |
| 3093 | RAB3GAP1 |
| 3094 | HMGA1 |
| 3095 | TCOF1 |
| 3096 | FGF9 |
| 3097 | AXIN2 |
| 3098 | CLNS1A |
| 3099 | CDK13 |
| 3100 | GYG1 |
| 3101 | PPP3CC |
| 3102 | PNP |
| 3103 | RELB |
| 3104 | AVPR1A |
| 3105 | P1 |
| 3106 | LAMA2 |
| 3107 | NR4A1 |
| 3108 | HCLS1 |
| 3109 | REL |
| 3110 | GADD45A |
| 3111 | PVRL1 |
| 3112 | STAT2 |
| 3113 | COPA |
| 3114 | ATP2B4 |
| 3115 | ILF2 |
| 3116 | MAP2K4 |
| 3117 | MMADHC |
| 3118 | TMPO |
| 3119 | FKBP9 |
| 3120 | ANAPC5 |
| 3121 | CTR9 |
| 3122 | THRAP3 |
| 3123 | RAB7L1 |
| 3124 | CAV1 |
| 3125 | FCGR2A |
| 3126 | BACE1 |
| 3127 | NDUFS3 |
| 3128 | TMEM173 |
| 3129 | SENP2 |
| 3130 | DCX |
| 3131 | PSMC6 |
| 3132 | RAB1A |
| 3133 | LYN |
| 3134 | SDHA |
| 3135 | POT1 |
| 3136 | ATRN |
| 3137 | L3MBTL2 |
| 3138 | PSMD6 |
| 3139 | ERG |
| 3140 | UBE2L3 |
| 3141 | MEG3 |
| 3142 | STAG1 |
| 3143 | GTPBP4 |
| 3144 | EFNA5 |
| 3145 | AXIN1 |
| 3146 | YIPF5 |
| 3147 | SPIN1 |
| 3148 | RHOBTB3 |
| 3149 | SHMT1 |
| 3150 | GCN1L1 |
| 3151 | TYRO3 |
| 3152 | CLTC |
| 3153 | CENPL |
| 3154 | SPRY2 |
| 3155 | HEY2 |
| 3156 | PPP4R1 |
| 3157 | SMG5 |
| 3158 | PPP4C |
| 3159 | OSBPL8 |
| 3160 | WFS1 |
| 3161 | CA12 |
| 3162 | CTSC |
| 3163 | PRKACA |
| 3164 | TINF2 |
| 3165 | REST |
| 3166 | SURF4 |
| 3167 | MZT2B |
| 3168 | TJP2 |
| 3169 | FAM48A |
| 3170 | ARHGAP21 |
| 3171 | LBR |
| 3172 | SHH |
| 3173 | AMOT |
| 3174 | IARS2 |
| 3175 | AVP |
| 3176 | IL6ST |
| 3177 | C18orf1 |
| 3178 | CHAF1A |
| 3179 | TNFSF11 |
| 3180 | UPF1 |
| 3181 | BAT3 |
| 3182 | C16orf57 |
| 3183 | CD247 |
| 3184 | EVL |
| 3185 | CCDC85B |
| 3186 | SH3BGRL3 |
| 3187 | OCRL |
| 3188 | LMNB2 |
| 3189 | P2RX7 |
| 3190 | DLG1 |
| 3191 | LIMK2 |
| 3192 | KRAS |
| 3193 | CEP57 |
| 3194 | CBX8 |
| 3195 | PSMC3 |
| 3196 | MAN2A1 |
| 3197 | FERMT2 |
| 3198 | SMC3 |
| 3199 | SMN1 |
| 3200 | MAP3K4 |
| 3201 | IL15 |
| 3202 | NOS1 |
| 3203 | SH3GL1 |
| 3204 | CSF1 |
| 3205 | CLN6 |
| 3206 | SLC25A4 |
| 3207 | CALM1 |
| 3208 | SCN5A |
| 3209 | ARHGEF10 |
| 3210 | TNPO3 |
| 3211 | P2RY1 |
| 3212 | THBS1 |
| 3213 | CAPZA2 |
| 3214 | PHLDB1 |
| 3215 | THBD |
| 3216 | SAE1 |
| 3217 | NCOA6 |
| 3218 | OS9 |
| 3219 | ARPC1A |
| 3220 | CC2D2A |
| 3221 | TRADD |
| 3222 | TMEM123 |
| 3223 | MAPKAP1 |
| 3224 | EBNA1BP2 |
| 3225 | POMGNT1 |
| 3226 | CD46 |
| 3227 | COL4A1 |
| 3228 | GPC4 |
| 3229 | MAP2K3 |
| 3230 | B2M |
| 3231 | SALL1 |
| 3232 | PRKCI |
| 3233 | SMARCC2 |
| 3234 | PSMD9 |
| 3235 | SOCS3 |
| 3236 | TFE3 |
| 3237 | JMJD6 |
| 3238 | SLC25A46 |
| 3239 | FAT1 |
| 3240 | FTL |
| 3241 | U2AF2 |
| 3242 | FASLG |
| 3243 | TNFRSF10B |
| 3244 | VPS4B |
| 3245 | IDE |
| 3246 | SF3A2 |
| 3247 | LIMK1 |
| 3248 | FAM36A |
| 3249 | MYBBP1A |
| 3250 | TBC1D20 |
| 3251 | CENPF |
| 3252 | ATF4 |
| 3253 | FAM38B |
| 3254 | ATP6V1E1 |
| 3255 | UMPS |
| 3256 | PRDX6 |
| 3257 | CDC20 |
| 3258 | PRKD2 |
| 3259 | TMEM66 |
| 3260 | PTCH1 |
| 3261 | FER |
| 3262 | NPLOC4 |
| 3263 | CAMK2G |
| 3264 | SCAR5 |
| 3265 | XPC |
| 3266 | RAB21 |
| 3267 | SDC1 |
| 3268 | LCN2 |
| 3269 | MLF2 |
| 3270 | TBRG4 |
| 3271 | CANT1 |
| 3272 | H3F3A |
| 3273 | PRKCE |
| 3274 | PRKCQ |
| 3275 | RNF185 |
| 3276 | RNF19A |
| 3277 | BAG4 |
| 3278 | EIF2A |
| 3279 | CD44 |
| 3280 | USP9X |
| 3281 | PRKCG |
| 3282 | PRKD1 |
| 3283 | FBXO7 |
| 3284 | GPD1L |
| 3285 | ATXN1 |
| 3286 | SART1 |
| 3287 | DAB2 |
| 3288 | SMG6 |
| 3289 | GOLM1 |
| 3290 | ARNT |
| 3291 | ARGLU1 |
| 3292 | COG6 |
| 3293 | MAP1LC3B |
| 3294 | SREBF2 |
| 3295 | C20orf30 |
| 3296 | ORC1L |
| 3297 | PSMD3 |
| 3298 | DBP |
| 3299 | SLC12A6 |
| 3300 | PRKAR2B |
| 3301 | CASP9 |
| 3302 | SMARCE1 |
| 3303 | CDC5L |
| 3304 | CDC23 |
| 3305 | FGFR2 |
| 3306 | MYADM |
| 3307 | PRKACB |
| 3308 | C1R |
| 3309 | CARD9 |
| 3310 | ORF1 |
| 3311 | CHKA |
| 3312 | STIP1 |
| 3313 | SALL4 |
| 3314 | MAST1 |
| 3315 | EIF4A3 |
| 3316 | GOLPH3 |
| 3317 | CAPZA1 |
| 3318 | TFRC |
| 3319 | FOXA1 |
| 3320 | CEP63 |
| 3321 | LARS |
| 3322 | MAP2K2 |
| 3323 | REEP5 |
| 3324 | PRKAG2 |
| 3325 | P4HA1 |
| 3326 | SCAR2 |
| 3327 | CAV3 |
| 3328 | SRF |
| 3329 | ERF |
| 3330 | CARKD |
| 3331 | C20orf43 |
| 3332 | FAR1 |
| 3333 | APBB1 |
| 3334 | ANXA5 |
| 3335 | CASK |
| 3336 | PPIG |
| 3337 | MLL2 |
| 3338 | LARP1 |
| 3339 | KRT31 |
| 3340 | GARS |
| 3341 | RFXANK |
| 3342 | FAM168B |
| 3343 | CCNL1 |
| 3344 | LCP1 |
| 3345 | HMGN3 |
| 3346 | CLIP3 |
| 3347 | DDX1 |
| 3348 | LAS1L |
| 3349 | CAV2 |
| 3350 | CCNB1 |
| 3351 | ARID5B |
| 3352 | ARIH1 |
| 3353 | RDBP |
| 3354 | SDCBP |
| 3355 | IFT140 |
| 3356 | GBF1 |
| 3357 | NDUFA4 |
| 3358 | B3GAT3 |
| 3359 | PSMD10 |
| 3360 | JMJD1C |
| 3361 | LEF1 |
| 3362 | PODXL |
| 3363 | ITPR2 |
| 3364 | CBX3 |
| 3365 | POMT2 |
| 3366 | ATXN2L |
| 3367 | CYB5B |
| 3368 | C20orf3 |
| 3369 | RPL24 |
| 3370 | MAT2B |
| 3371 | SAP130 |
| 3372 | ITPR3 |
| 3373 | NPC2 |
| 3374 | PSMD14 |
| 3375 | CALCA |
| 3376 | PAIP2 |
| 3377 | MATR3 |
| 3378 | VDR |
| 3379 | DNAJC3 |
| 3380 | DAG1 |
| 3381 | LCK |
| 3382 | ARL2 |
| 3383 | EFNB2 |
| 3384 | GATA3 |
| 3385 | GADD45GIP1 |
| 3386 | CASR |
| 3387 | PTCD3 |
| 3388 | FHIT |
| 3389 | LMBRD1 |
| 3390 | F2RL1 |
| 3391 | SDC2 |
| 3392 | PICK1 |
| 3393 | MAF |
| 3394 | USP24 |
| 3395 | PRKG1 |
| 3396 | LYST |
| 3397 | MUC1 |
| 3398 | CYP1A1 |
| 3399 | ELANE |
| 3400 | DNAJC10 |
| 3401 | PLSCR1 |
| 3402 | FCER1G |
| 3403 | FGB |
| 3404 | FGA |
| 3405 | LDB1 |
| 3406 | LCP2 |
| 3407 | PRICKLE2 |
| 3408 | SRP54 |
| 3409 | FBXO18 |
| 3410 | CAMTA1 |
| 3411 | LDLR |
| 3412 | SNRPB |
| 3413 | C3 |
| 3414 | DNAJC5 |
| 3415 | NOP10 |
| 3416 | DDX17 |
| 3417 | SMC1A |
| 3418 | PCSK9 |
| 3419 | EFTUD2 |
| 3420 | MEOX2 |
| 3421 | PPP2R2A |
| 3422 | MKI67 |
| 3423 | NUP54 |
| 3424 | KCNQ1 |
| 3425 | GAA |
| 3426 | TOMM70A |
| 3427 | CKAP2 |
| 3428 | CA2 |
| 3429 | IL13 |
| 3430 | GHITM |
| 3431 | NDN |
| 3432 | PTBP2 |
| 3433 | MYOC |
| 3434 | AZU1 |
| 3435 | DAB2IP |
| 3436 | C19orf62 |
| 3437 | DNAJC7 |
| 3438 | PSORS1 |
| 3439 | TRAF3 |
| 3440 | PGRMC1 |
| 3441 | GHRL |
| 3442 | HIPK2 |
| 3443 | DACT1 |
| 3444 | CASP2 |
| 3445 | MARK3 |
| 3446 | PPME1 |
| 3447 | PSAT1 |
| 3448 | COX15 |
| 3449 | FGFR3 |
| 3450 | SDCCAG8 |
| 3451 | DACH1 |
| 3452 | MYO7A |
| 3453 | MYO6 |
| 3454 | FBXL4 |
| 3455 | FASN |
| 3456 | PSMD13 |
| 3457 | PSMD2 |
| 3458 | SPECC1L |
| 3459 | PBX3 |
| 3460 | EREG |
| 3461 | C22orf28 |
| 3462 | UBE2N |
| 3463 | PC |
| 3464 | HLA-B |
| 3465 | TMF1 |
| 3466 | KIF5B |
| 3467 | ENPP1 |
| 3468 | XDH |
| 3469 | FAT4 |
| 3470 | PRX |
| 3471 | DNAJB11 |
| 3472 | RPL23A |
| 3473 | TBX2 |
| 3474 | TNFRSF1A |
| 3475 | MYO19 |
| 3476 | MIP |
| 3477 | C2orf86 |
| 3478 | ARRB2 |
| 3479 | IL12B |
| 3480 | BIRC6 |
| 3481 | DDX6 |
| 3482 | LIMCH1 |
| 3483 | MPI |
| 3484 | KIF7 |
| 3485 | CNN3 |
| 3486 | FZD4 |
| 3487 | IDDM12 |
| 3488 | MOAP1 |
| 3489 | PRKAR1A |
| 3490 | SESN2 |
| 3491 | LPL |
| 3492 | DDOST |
| 3493 | PDCD6IP |
| 3494 | RPL6 |
| 3495 | H2AFZ |
| 3496 | TRIB3 |
| 3497 | ID3 |
| 3498 | NISCH |
| 3499 | CKS1B |
| 3500 | NR4A3 |
| 3501 | FADD |
| 3502 | GALT |
| 3503 | TRIM13 |
| 3504 | DAPK1 |
| 3505 | PSMB4 |
| 3506 | SMPD1 |
| 3507 | SRD5A1 |
| 3508 | TREM2 |
| 3509 | MTUS1 |
| 3510 | DLST |
| 3511 | CYBA |
| 3512 | GRIK2 |
| 3513 | MAT2A |
| 3514 | DNAH11 |
| 3515 | HNRNPF |
| 3516 | ST6GAL1 |
| 3517 | MAGED1 |
| 3518 | HSPB1 |
| 3519 | DDR2 |
| 3520 | RDX |
| 3521 | CALU |
| 3522 | RALA |
| 3523 | HSPA5 |
| 3524 | NMB |
| 3525 | MAPK1 |
| 3526 | GNL3 |
| 3527 | IBTK |
| 3528 | CBS |
| 3529 | YARS |
| 3530 | TTC19 |
| 3531 | TSPAN3 |
